# Artificial Intelligence–Enabled Analysis of Statin-Related Topics and Sentiments on Social Media

**DOI:** 10.1001/jamanetworkopen.2023.9747

**Published:** 2023-04-24

**Authors:** Sulaiman Somani, Marieke Meija van Buchem, Ashish Sarraju, Tina Hernandez-Boussard, Fatima Rodriguez

**Affiliations:** 1 Department of Medicine, Stanford University, Stanford, California; 2 Department of Information Technology & Digital Innovation, Leiden University Medical Center (LUMC), Leiden, the Netherlands; 3 CAIRELab, LUMC, Leiden, the Netherlands; 4 Department of Biomedical Data Science, Stanford University, Stanford, California; 5 Department of Cardiovascular Medicine, Cleveland Clinic, Cleveland, Ohio; 6 Department of Surgery, Stanford University School of Medicine, Stanford, California; 7 Division of Cardiovascular Medicine and Cardiovascular Institute, Stanford University, Stanford, California

## Abstract

**Importance:**

Despite compelling evidence that statins are safe, are generally well tolerated, and reduce cardiovascular events, statins are underused even in patients with the highest risk. Social media may provide contemporary insights into public perceptions about statins.

**Objective:**

To characterize and classify public perceptions about statins that were gleaned from more than a decade of statin-related discussions on Reddit, a widely used social media platform.

**Design, Setting, and Participants:**

This qualitative study analyzed all statin-related discussions on the social media platform that were dated between January 1, 2009, and July 12, 2022. Statin- and cholesterol-focused communities, were identified to create a list of statin-related discussions. An artificial intelligence (AI) pipeline was developed to cluster these discussions into specific topics and overarching thematic groups. The pipeline consisted of a semisupervised natural language processing model (BERT [Bidirectional Encoder Representations from Transformers]), a dimensionality reduction technique, and a clustering algorithm. The sentiment for each discussion was labeled as *positive*, *neutral*, or *negative* using a pretrained BERT model.

**Exposures:**

Statin-related posts and comments containing the terms *statin* and *cholesterol*.

**Main Outcomes and Measures:**

Statin-related topics and thematic groups.

**Results:**

A total of 10 233 unique statin-related discussions (961 posts and 9272 comments) from 5188 unique authors were identified. The number of statin-related discussions increased by a mean (SD) of 32.9% (41.1%) per year. A total of 100 discussion topics were identified and were classified into 6 overarching thematic groups: (1) ketogenic diets, diabetes, supplements, and statins; (2) statin adverse effects; (3) statin hesitancy; (4) clinical trial appraisals; (5) pharmaceutical industry bias and statins; and (6) red yeast rice and statins. The sentiment analysis revealed that most discussions had a neutral (66.6%) or negative (30.8%) sentiment.

**Conclusions and Relevance:**

Results of this study demonstrated the potential of an AI approach to analyze large, contemporary, publicly available social media data and generate insights into public perceptions about statins. This information may help guide strategies for addressing barriers to statin use and adherence.

## Key Points

**Question:**

What are public perceptions of statins as expressed on a widely used social media platform?

**Findings:**

In this qualitative study of 10 233 unique statin-related discussions, an artificial intelligence (AI) pipeline was developed to analyze these discussions, which were automatically categorized into 100 topics and 6 thematic groups (ketogenic diets, diabetes, supplements, and statins; statin adverse effects; statin hesitancy; clinical trial appraisals; pharmaceutical industry bias and statins; and red yeast rice and statins). Sentiment analysis of these discussions showed that most of them had a neutral or negative sentiment.

**Meaning:**

Findings from this study suggest that AI-enabled analysis of large, contemporary social media data may generate insights into public perceptions and help guide strategies for addressing barriers to statin use and adherence.

## Teaser

This qualitative study evaluates user-generated, publicly available posts and comments about statin-related topics: ketogenic diets, diabetes, and supplements; statin adverse effects; statin hesitancy; clinical trial appraisals; pharmaceutical industry bias; and red yeast rice.

## Introduction

Statins conclusively reduce morbidity and mortality from atherosclerotic cardiovascular disease (ASCVD), the number one cause of death worldwide.^1,2^ Thus, statins are a cornerstone of the treatment and prevention of ASCVD across contemporary clinical practice guidelines.^3^ Statins are one of the most commonly prescribed medications in the US, making up nearly 17% of all prescription pharmaceuticals in the past decade, in part because of their widespread availability, low cost, high degree of effectiveness, and breadth of clinical indications.^4,5^ However, despite their well-established benefits and safety, statin use remains suboptimal in high-risk individuals with guideline-recommended clinical indications.^6^

Understanding the reasons for statin underuse, including patient-level perspectives, is crucial to guiding public health and implementation efforts toward ASCVD prevention and treatment. While barriers to statin use have been explored using targeted surveys and focus groups, these may not be widely generalizable.^7,8,9^ Social media platforms have become a promising avenue to understand and glean public views on health outside of the health care setting.^10,11^ Such platforms are used for sharing personal stories, soliciting informal opinions, or sparking discussions about any topics. These platforms can rapidly disseminate information or misinformation through noncurated, transparent peer discussions.^12^ For example, a study analyzed more than 11 000 social media posts and manually annotated them to understand belief patterns regarding statins, many of which reflected established concerns about their use, even in statin nonusers.^13^

A social media platform that has steadily gained popularity for health-related purposes over the past years is Reddit, a discussion-based platform whose users can post questions, comments, and topics on a wide range of areas, including statins. The platform is free, has 52 million daily active users and approximately 430 million monthly users, and gets more than 30 billion views every month.^14^ Given its widespread use, this platform may provide large-scale data on patient views on statins that could be analyzed for novel insights and for misinformation that may affect statin adherence.^15^

Manual analysis of social media data has limited feasibility given the speed and volume with which these data evolve over time. Artificial intelligence (AI) methods may facilitate the analysis and interpretation of these valuable patient-generated data that may influence health behaviors. Natural language processing (NLP) is a form of AI that ingests and interprets large volumes of textual data to glean insights and that can make actionable predictions on new data.^16,17^ This study aimed to characterize and classify public perceptions about statins gleaned from more than a decade of statin-related discussions on Reddit.

## Methods

The Stanford University Institutional Review Board deemed this qualitative study exempt from ethical review and the requirement for informed consent since it did not involve human participants. We followed the Standards for Reporting Qualitative Research (SRQR) reporting guideline.^18^

### Data Set and Search

Reddit was used as the data source for this study.^19^ Data were collected between January 1, 2009, and July 12, 2022.

The social media platform is composed of different communities, which use the r/ prefix and are focused on specific topics (eg, r/gaming, r/worldnews, r/keto, and r/statins). Users may interact with the platform by creating a post to initiate a new discussion thread or by commenting on other users’ posts as part of discussions. Most communities, including all posts and comments, are openly or publicly accessible and visible and thus require no account registration with the social media platform.

To create a list of statin-related discussions on this social media platform, we identified relevant communities by entering the words *statin* and *cholesterol* in the platform search engine and keeping those communities that were recommended by the search engine for both words. Across these communities, we used an application programming interface called Pushshift to search all of the posts and comments for case-insensitive matching on the word *statin* and the generic or brand names for specific statins: atorvastatin, lipitor, rosuvastatin, crestor, pitavastatin, livalo, zypitamag, simvastatin, zocor, pravastatin, pravachol, lovastatin, altoprev, fluvastatin, and lescol (eFigure 1 in Supplement 1).^20^

### Topic Modeling

Raw text that was collected from the social media platform was preprocessed to prepare it for automatic analysis. We used BERTopic, a state-of-the-art NLP technique that leverages the strength of BERT (Bidirectional Encoder Representations from Transformers) models to perform topic modeling to identify topics of discussion about statins. Briefly, BERTopic first embeds documents using a sentence-level BERT model, called Sentence-BERT, and applies an unsupervised machine learning technique called UMAP (Uniform Manifold Approximation and Projection) to simplify this representation.^21^ The all-MiniLM-L6-v2 pretrained model was specifically chosen for the data set given its applicability for the social media platform and scientific content since it was already trained on more than 600 million posts and S2ORC, a data set containing more than 12.8 million papers in the field of medicine, among many other language data sets.^22^

Topics were identified by spectral clustering, an algorithm for grouping similar discussions together into topics. Clustering performance was measured using 2 metrics: Silhouette coefficient and Davies-Bouldin index.^23,24^ The Silhouette coefficient measures the similarity of a discussion to its own topic (cohesion) compared with its similarity to other topics (separation). A Silhouette coefficient (range, −1 to 1) that is closer to 1 indicates better performance. The Davies-Bouldin index takes a more global approach and compares the average similarity of each topic to its most similar topic. A Davies-Bouldin index score (range, 0-1) that is closer to 0 indicates more dispersed and less similar topics.

Since these topics can be granular and may overlap substantially, we performed a subsequent clustering analysis on a mathematical representation of these topics to find overarching themes of discussion (groups). Sensitivity analyses that maximized the Silhouette coefficient and Davies-Bouldin index were performed to identify the optimal number of groups. Full details on preprocessing, model selection, dimensionality reduction (UMAP), topic and group clustering, and sensitivity analyses are provided in the eMethods in Supplement 1.

### Sentiment Analysis

Sentiment analysis is a technique for identifying and extracting subjective information from text documents. A common form of sentiment analysis is to classify the tone of text documents into distinct categories, such as positive (eg, “I love statins!”) or negative (eg, “I hate statins!”).^25,26,27^

To assess the sentiments for each post, we used a pretrained BERT model, called RoBERTa, that was trained on social media posts.^21^ RoBERTa offers multiclass labels (ie, positive, neutral, or negative classification of text) and has been used in recent studies investigating health care problems using data from social media.^28,29,30,31^ To quantify how sentiments varied across topics and groups, we transformed sentiment labels to scores: from negative to −1, neutral to 0, and positive to 1.^32^ Full details on algorithm and model choice, input data handling, and output transformation are provided in the eMethods in Supplement 1.

### Statistical Analysis

We described discussion characteristics using mean and SD. Data analysis was performed from July to August 2022. Analysis was performed using the Python programming language, version 3.7.3 (Python Software Foundation) and multiple key libraries: scikit-learn, version 1.1.1; BERTopic, version 0.11.0; transformers, version 4.20.1; and matplotlib, version 3.5.2. Code that was developed for topic modeling and analysis is available on https://www.github.com/sssomani/statins_reddit.

## Results

A total of 19 communities that contained both search terms *statin* and *cholesterol* were identified from a candidate list of 75 communities. From these 19 communities, a total of 10 233 unique statin-related posts and comments were curated, which included 961 unique statin-related posts and 9272 unique statin-related comments from 5188 unique authors (Table 1). Posts were longer in mean (SD) number of characters than comments (1792.9 [2693.5] vs 839.9 [1122.1]). Most comments and posts were retrieved from matching the word *statin* (74.0%). *Lipitor* (12.7%) and *atorvastatin* (4.2%) were the second and third most common search terms, followed by *Crestor* (3.8%), *simvastatin* (1.8%), and *rosuvastatin* (1.7%). The communities that most frequently contained these posts and comments were r/keto (23.1%) and r/Cholesterol (21.3%), followed by r/diabetes (8.6%) and r/science (6.9%).

**Table 1.** Post and Comment Summary Statistics

Characteristic, category	No. (%)		
	All discussions	Comments	Posts
No. of discussions scraped	10 233	9272	961
No. of characters, mean (SD)	929.4 (1377.9)	839.9 (1122.1)	1792.9 (2693.5)
Unique authors	5188 (50.7)	4700 (50.7)	779 (81.1)
Search words			
Statin	7571 (74.0)	6891 (74.3)	680 (70.8)
Lipitor	1295 (12.7)	1218 (13.1)	77 (8.0)
Atorvastatin	434 (4.2)	367 (4.0)	67 (7.0)
Crestor	389 (3.8)	357 (3.9)	32 (3.3)
Simvastatin	181 (1.8)	138 (1.5)	43 (4.5)
Rosuvastatin	177 (1.7)	141 (1.5)	36 (3.7)
Lovastatin	73 (0.7)	65 (0.7)	8 (0.8)
Pravastatin	71 (0.7)	56 (0.6)	15 (1.6)
Zocor	21 (0.2)	19 (0.2)	2 (0.2)
Pitavastatin	10 (0.1)	10 (0.1)	0
Fluvastatin	5 (0)	5 (0.1)	0
Livalo	4 (0)	3 (0)	1 (0.1)
Pravachol	2 (0)	2 (0)	0
Community			
r/keto	2364 (23.1)	2051 (22.1)	313 (32.6)
r/Cholesterol	2182 (21.3)	1875 (20.2)	307 (31.9)
r/diabetes	879 (8.6)	748 (8.1)	131 (13.6)
r/science	706 (6.9)	704 (7.6)	2 (0.2)
r/ketoscience	560 (5.5)	504 (5.4)	56 (5.8)
r/nutrition	503 (4.9)	486 (5.2)	17 (1.8)
r/ScientificNutrition	446 (4.4)	426 (4.6)	20 (2.1)
r/news	443 (4.3)	443 (4.8)	0
r/todayilearned	381 (3.7)	380 (4.1)	1 (0.1)
r/conspiracy	375 (3.7)	355 (3.8)	20 (2.1)
r/Supplements	367 (3.6)	326 (3.5)	41 (4.3)
r/Health	238 (2.3)	230 (2.5)	8 (0.8)
r/PlantBasedDiet	230 (2.2)	208 (2.2)	22 (2.3)
r/askscience	165 (1.6)	154 (1.7)	11 (1.1)
r/COVID19	136 (1.3)	135 (1.5)	1 (0.1)
r/Paleo	112 (1.1)	105 (1.1)	7 (0.7)
r/longevity	75 (0.7)	73 (0.8)	2 (0.2)
r/skeptic	68 (0.7)	68 (0.7)	0
r/StopUsingStatins	3 (0)	1 (0)	2 (0.2)

**Figure 1.**
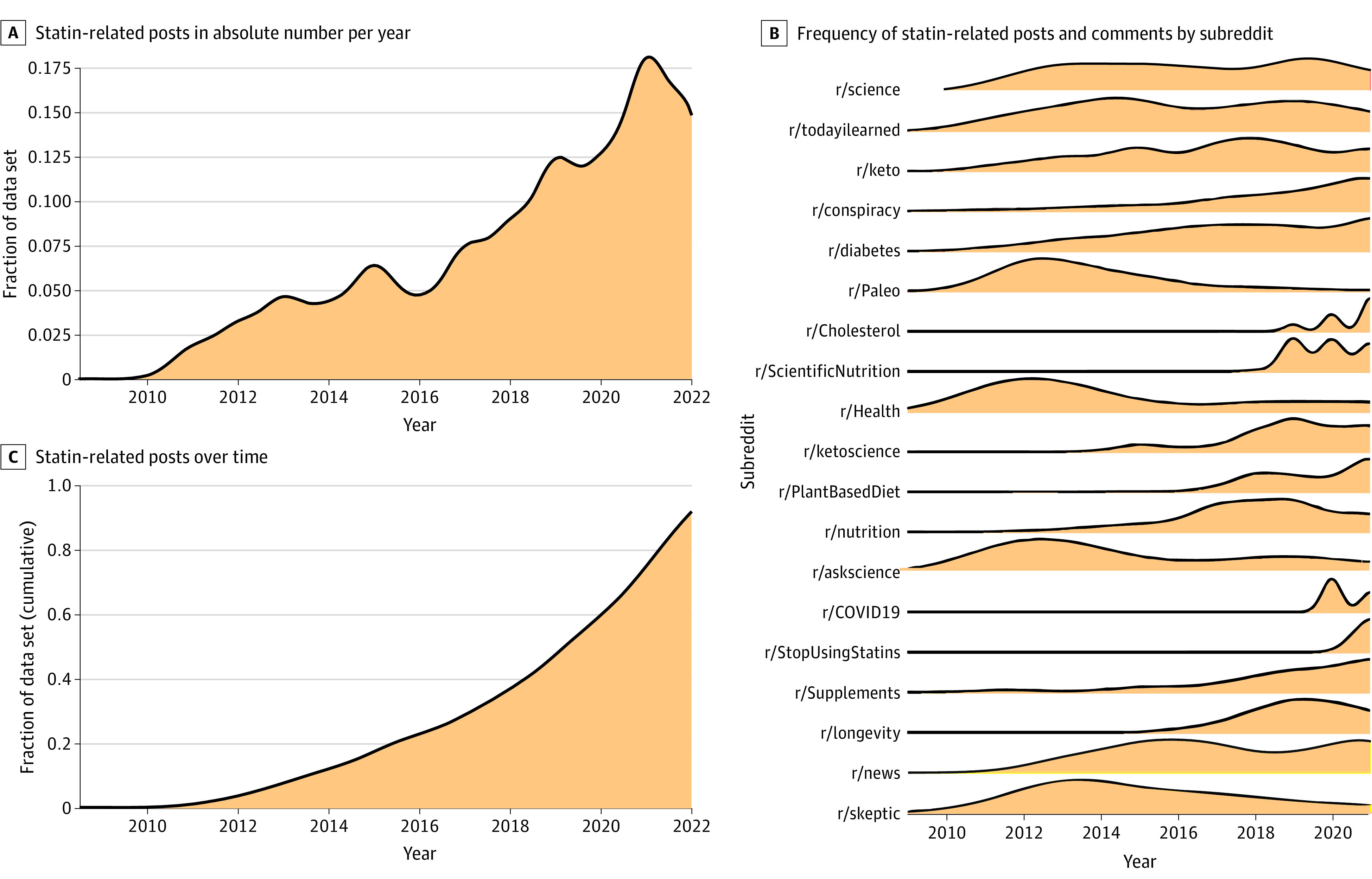
Statin-Related Posts and Comments Over Time When mapped over time, the number of statin-related posts in the data set increased in absolute number per year (A) and cumulatively over time (C). The frequency of posts and comments varied by community over time (B).

A total of 779 unique users (81.1%) authored all posts, and 4700 unique users (50.7%) authored all comments (Table 1). Most authors had between 1 and 5 posts (94.6%) (eFigure 2 in Supplement 1). The number of statin-related discussions increased by a mean (SD) of 32.9% (41.1%) per year (Figure 1A). However, the number of statin-related discussions on certain communities increased yearly (eg, r/Supplements, r/conspiracy, and r/diabetes) but decreased in others (such as r/Paleo and r/skeptic) (Figure 1B).

**Figure 2.**
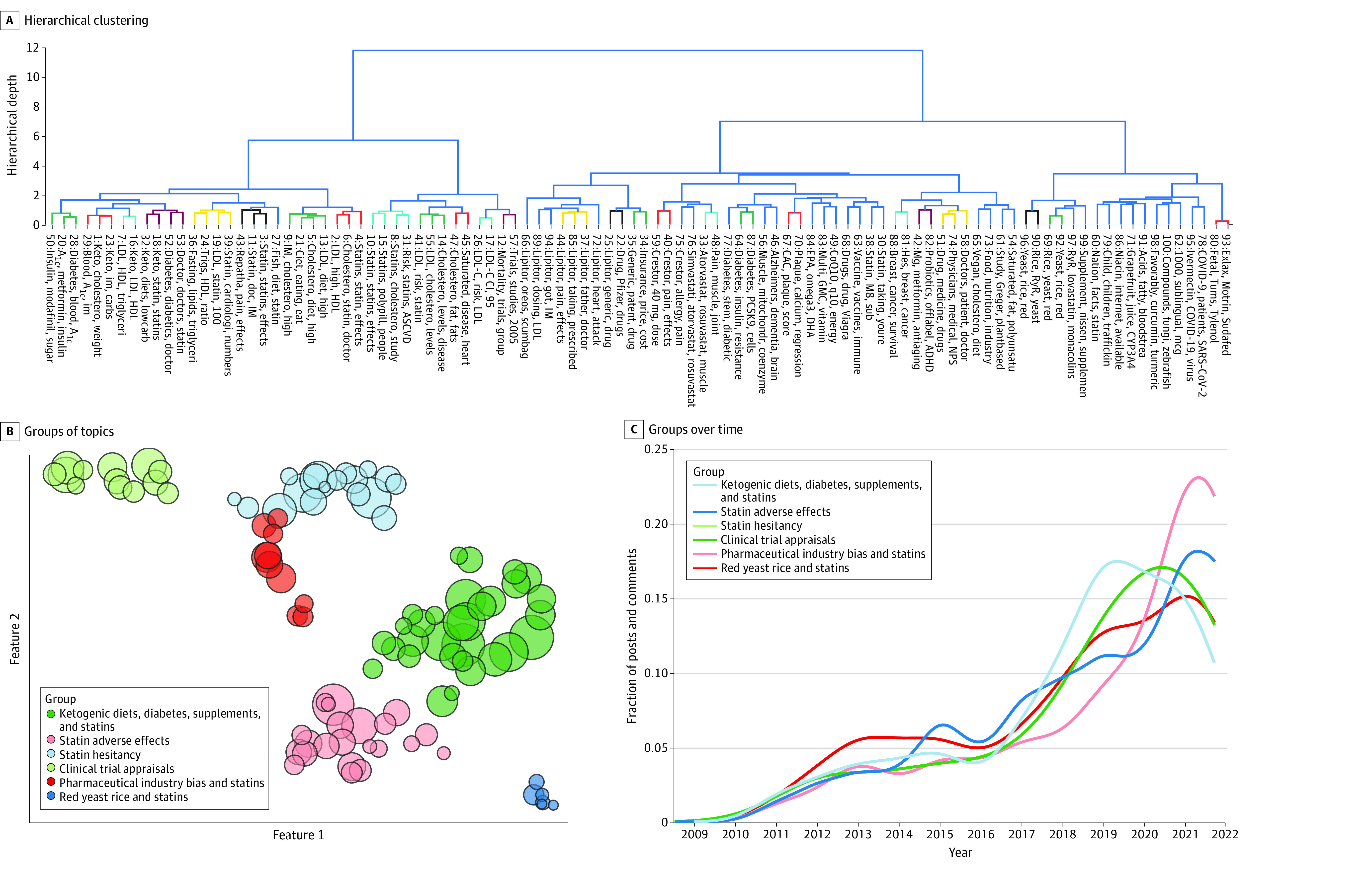
Topic Modeling Hierarchical (A) and spatial (B) representations of the 100 extracted topics (columns in A; circles in B) and 6 overarching groups are shown. In panel A, the y-axis represents the depth of the hierarchical tree corresponding to each node in the tree. The size of each topic represents the relative number of discussions grouped in that topic. Changes in the number of discussions for each of the 6 groups over time are shown in panel C. The x- and y-axes in panel B represent the 2 Uniform Manifold Approximation and Projection axes that were dimensionally reduced to allow for topic visualization. ADHD indicates attention deficit/hyperactivity disorder; ASCVD, atherosclerotic cardiovascular disease; CAC, coronary artery calcium; coQ10, coenzyme Q10; CYP3A4, cytochrome P450 3A4; DHA, docosahexaenoic acid; doc, doctor; EPA, eicosapentaenoic acid; HDL, high-density lipoprotein; IM, I am; hes, he is; LDL, low-density lipoprotein; LDL-C, low-density lipoprotein cholesterol; M8, mate; NPS, nurse practitioner; RyR, ryanodine receptor; trigs, triglycerides.

A total of 100 topics of statin-related discussions were identified from the data set (eTable 1 in Supplement 1), with a performance Silhouette coefficient of 0.013 and a Davies-Bouldin index of 4.27. The 3 most common topics were elevated low-density lipoprotein cholesterol (LDL-C) when on a ketogenic diet (topic 1), advice and statin experience solicitation with changes in lipid panels (topic 2), and anecdotal perspectives on statin efficacy and adverse effects (topic 3). Other topics included adverse effects from statins (eg, topics 33, 39, 43, and 44); statin trial data and possible industry bias in their outcomes (eg, topics 22, 34, and 68); lifestyle alternatives, such as supplements (eg, topic 84); red yeast rice (eg, topic 69); dietary changes (eg, topics 5 and 65); improving outcomes with COVID-19 (eg, topics 63, 78, and 95); the interplay between coronary artery calcium scores and role of statins (eg, topic 67); and attitudes about statin development that used fetal stem cells (eg, topic 80). Hierarchical representation of these topics is shown in Figure 2A. Changes over time in the number of discussions per topic are presented in eFigure 3 in Supplement 1.

Six overarching groups (Figure 2B) from these 100 topics were identified after sensitivity analysis, maximizing the Silhouette coefficient and Davies-Bouldin index (eFigure 4 in Supplement 1). These groups were (1) ketogenic diets, diabetes, supplements, and statins; (2) statin adverse effects; (3) statin hesitancy; (4) clinical trial appraisals; (5) pharmaceutical industry bias and statins; and (6) red yeast rice and statins (Table 2). Temporal patterns in these thematic groups are shown in Figure 2C.

**Table 2.** Overview of Groups of Topics With Example Text

Group	Topic No.	No.		Description	Example text
		Posts	Comments		
1	1, 2, 5, 6, 7, 9, 13, 16, 18, 19, 20, 21, 23, 24, 27, 28, 29, 32, 36, 42, 50, 52, 53, 58, 67, 73, 77, 81, 83, 88, 91	691	3497	Ketogenic diets, diabetes, supplements, and statins	“Why is my cholesterol so high when I lost weight was working out had no fast food and my BP is good. I’ve seen it as low as 96/70 but it varies from 105/70 TO 120/80 while at home. Do you think I can fix this naturally?”
					“The rise of the calcium scan should make the decision easier. If you have a low calcium score then you probably don’t need it. The higher the score the more obvious you do need the statin because your cholesterol is calcifying. Also, baseline scans will help know if you are laying down an unusual amount of calcified plaque each year which would also argue for long term statin use.”
					“High LDL - 9 months of keto. My doctor is eager for me to go back on statin medication. I stopped taking it in 2019 due to side effects. I started keto in October 2019 and am now 45lbs lighter (34yo male). In October 2019 my number were: LDL: 181 HDL: 33 Triglycerides: 332. In June 2020 my numbers are: LDL: 256 HDL: 40 Triglycerides: 107. My doctor is extremely concerned about the LDL and says it must come down. There is a lot of conflicting information out there regarding keto and cholesterol. I’m hoping to receive some guidance and sources from the gurus in the subject.”
2	3, 11, 30, 33, 37, 39, 40, 43, 44, 48, 59, 60, 62, 66, 75, 76, 80, 85, 89, 93, 94, 96	102	1768	Statin adverse effects	“I will never take a statin again.”
					“That’s very consistent with statins. Top 3 adverse events with the class are muscle aches (myalgia), joint aches ([arthralgia]), and fatigue. Call your doctor and see what they say. Typically, the recommendation is to take a 2-week holiday and then try a different statin at a lower dose.”
					“My endocrinologist wants me to go on Lipitor but after reading info with the prescription, muscle loss is a side effect. I have enough trouble meeting my protein goals as it is and take supplemental Mg and Potassium to stave off muscle cramps, so its going to stay on the shelf for now.”
3	4, 8, 10, 15, 31, 38, 41, 46, 49, 56, 63, 64, 70, 72, 86, 87, 95, 98	79	1775	Statin hesitancy: initiation, maintenance, and alternatives	“I’m not completely anti-statin, but I’m living proof that diet alone *can* change cholesterol drastically (see post above, or below if it got downvoted).”
					“I’m just mad at the fact you said statins aren’t healthy which is completely false in people using it for primary prevention or for diabetics….”
					“Given the sheer density of research performed on this remarkable spice [curcumin], it is no wonder that a growing number of studies have concluded that it compares favorably to a variety of conventional medications, including: * Lipitor/Atorvastatin (cholesterol medication)….”
4	12, 14, 17, 26, 45, 47, 54, 55, 57, 61, 65, 78, 84	73	1271	Clinical trial appraisals	“Large study finds lower risk of death from COVID-19 in statin users. Probably because the Statins will kill you first.”
					“People seem to think that statins are there to lower LDL-C. However, there are no trials to show statins work in that setting. Statins don’t really lower CVD events that much. It’s very controversial whether they lower all-cause mortality at all….”
					“Statins are lipid-lowering therapeutics with favorable anti-inflammatory profiles and have been proposed as an adjunct therapy for COVID-19. However, statins may increase the risk of SARS-CoV-2 viral entry by inducing ACE2 expression.”
5	22, 25, 34, 35, 51, 68, 71, 74, 79, 82	10	837	Pharmaceutical industry bias and statins	“The cost of drugs truly is crazy. I work in a pharmacy and look at the cash cost of a lot of things just out of curiosity. Atorvastatin (generic lipitor) at a moderate dosage is like $450/month cash price at my pharmacy.”
					“Uh oh, your cholesterol is high. Here’s some Lipitor ... and so on forever. Pharmaceutical companies are not charitable. They are corporations and they are in the business of symptom maintenance. Nothing more. No cures, only once a day pills. Doctors are not charities. They are businesses, and patients are customers.”
					“You might not find this, but there’s a lot of statins that can be taken with grapefruit juice.”
6	69, 90, 92, 97, 99, 100	6	124	Red yeast rice and statins	“red yeast rice is a statin, and statins should be avoided at all costs. If you have high cholesterol and also mind your diet, you may want to check LMHR communities and the cholesterol code. statins are basically mycotoxins and deplete you if fat soluble nutrients, like coQ10, vit D, K, A and E, and in all likelihood through these depletions worsen cardiovascular health.”
					“When they discovered statin drugs for cholesterol, you look back at ancient Chinese remedies for cardiovascular health and find fermented red yeast rice over 1000 years ago which can actually create statin compounds.”

Abbreviations: ACE2, angiotensin-converting enzyme 2; BP, blood pressure; coQ10, coenzyme Q10; CVD, cardiovascular disease; HDL, high-density lipoprotein; LDL, low-density lipoprotein; LDL-C, LDL cholesterol; LMHR, lean mass hyper-responder; vit, vitamin.

**Figure 3.**
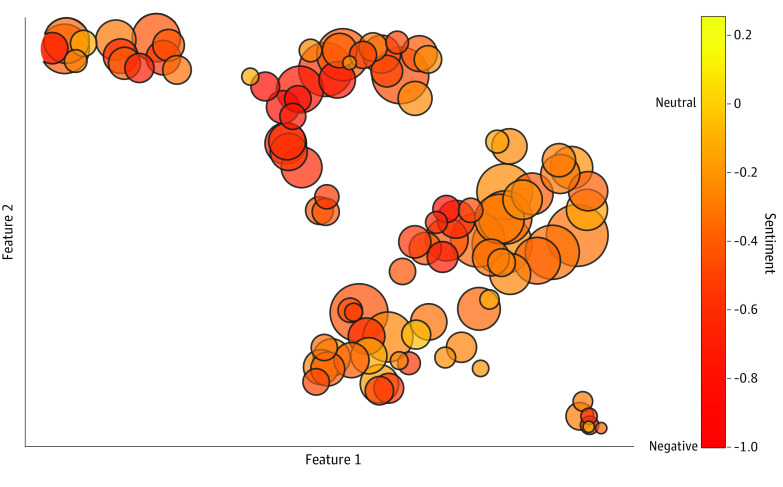
Sentiment Analysis Mean sentiment (color) across topics (circles) is shown. The size of each topic represents the relative number of discussions grouped in that topic. Mean sentiment scores that were close to −1 reflected a predominantly negative sentiment (red), close to 0 reflected an overall neutral sentiment (yellow), and close to 1 reflected an overall positive sentiment. Because no topics had a positive sentiment, the color map was truncated at 0.2 to allow for differentiation between negative and neutral sentiments. The x- and y-axes represent the 2 Uniform Manifold Approximation and Projection axes that were dimensionally reduced to allow for topic visualization.

Of the 10 233 discussions, 3151 had a negative (30.8%), 6815 had a neutral (66.6%), and 267 had a positive (2.6%) sentiment. Examples of discussions with a highly negative sentiment included the following: “So take a statin to decrease CVD risk ... but increase Alzheimer’s risk? What a mess!,” “And then statin kills you instead of covid,” and “Statins are poison. It’s a myth made up by the pharma industry obviously to make more $$$....” Examples of discussions with a highly positive sentiment included, “I love taking my simvastatin … it also cured my psoriasis,” “I sure do love me a statin,” and “‘I love Crestor, the taste is better.” The mean (SD) sentiment score across all discussions was considered to be neutral to negative (score, −0.28 [0.50]). Among topics, 2 had sentiment scores that were considered to be neutral (topics 62 and 100); all other topics had negative sentiments (Figure 3). None of the 6 groups represented a positive sentiment. Mean (SD) sentiment score by communities ranged from −0.52 (0.51) for r/conspiracy, which reflected a more negative sentiment, to −0.12 (0.35) for r/COVID19, which reflected a more neutral sentiment (eTable 2 in Supplement 1).

## Discussion

This qualitative study leveraged more than a decade of patient-generated data from a social media platform to uncover public beliefs and perceptions about statins. Artificial intelligence methods were used to analyze 10 233 unique discussions from 5188 unique authors, which increased over time. This approach identified 100 topics from the discussion that represented 6 thematic groups: (1) ketogenic diets, diabetes, supplements, and statins; (2) statin adverse effects; (3) statin hesitancy; (4) clinical trial appraisals; (5) pharmaceutical industry bias and statins; and (6) red yeast rice and statins. Sentiment analysis demonstrated that these discussions had a predominantly neutral to negative sentiment. These findings highlighted community perceptions and potentially modifiable barriers to statin use.

This study demonstrated the potential of AI to automate the extraction and analysis of social media data to understand public perceptions on statins. It complements and extends prior work that evaluated statin attitudes and beliefs using manual qualitative analyses of Twitter posts.^13^ Since topic prespecification can miss unexpected emerging ideas, the study’s AI-enabled algorithm semiautomatically organized discussions into topics and broader groups while categorizing the sentiments on these discussions. By efficiently extracting and interpreting large volumes of valuable social media data, AI offers the prospect of monitoring public sentiment continuously at scale.

The primary groups of discussion in this study align with findings in prior studies that assessed patient beliefs about statins. For example, the USAGE (Understanding Statin Use in America and Gaps in Patient Education) study and PALM (Patient and Provider Assessment of Lipid Management) Registry have identified patient perceptions and barriers to statin adherence.^6,7,33,34^ The present study corroborated some of these previous findings, uncovering similar reasons for statin hesitancy, including adverse effect profiles (eg, myalgias, increased risk of diabetes, and cognitive dysfunction; group 2), disbelief in the LDL-C hypothesis, preference for lifestyle alternatives (eg, dietary or supplementary modifications; groups 1, 3, and 6), and general disenfranchisement with health care (group 5). Furthermore, novel points of discourse were uncovered, such as the role of statins in improving COVID-19 outcomes, controversy regarding lifestyle improvement on a ketogenic diet but developing asymptomatic dyslipidemia, the use of the coronary artery calcium score as a convincing data point for initiating a statin, and hesitancy toward statins because of concerns that statins were made with fetal stem cells. Leveraging anonymous, noncurated, informal peer-to-peer discussions from public social media platforms, such as Reddit, may reveal topics and sentiments that may not be identified in formal targeted surveys or focus groups, clinical encounters, or clinical trial settings.

Sentiment analysis of social media posts and discussions about statins revealed a predominantly neutral to negative sentiment. Prior work has highlighted that bad publicity surrounding statins can affect patients’ medication adherence. For example, a Danish study found that unfavorable media coverage about statins was associated with a decrease in new statin users and an increase in statin discontinuation among current statin users within 1 year of the press coverage.^35^ More active public health efforts are needed to monitor health-related misinformation on readily accessible social media platforms.

Several examples of statin-related misinformation were identified, including distrust of the hypothesis that LDL-C has a causal association with heart disease (eg, “I think LDL is pretty much irrelevant. Your HDL and Triglycerides are far more important” [r/keto, topic 7]) and of the association between COVID-19 and statins (eg, “results imply the potential benefits of statin therapy in hospitalized subjects with COVID-19” [r/COVID19, topic 78]). While support for natural supplemental alternatives (eg, “Red yeast rice is a statin basically, by the way” [r/Cholesterol, topic 69]; “statins are basically mycotoxins and deplete you if [*sic*] fat soluble nutrients, like coQ10, vit D, K, A and E, and in all likelihood through these depletions worsen cardiovascular health” [r/Supplements, topic 90]) was powerful in this study across multiple thematic groups (1, 2, and 4), the recent SPORT (Supplements, Placebo, or Rosuvastatin) trial demonstrated a substantial decrease in LDL-C with low-dose rosuvastatin but not with common supplements compared with placebo.^36^ Such misperceptions posted on social media platforms can serve as seeds for the spread of misinformation. For example, discussions on the social media platform used in this study are easily accessible since the platform does not require users to have an account to see content and are highly presented on search engines, with some individuals even using this platform as their default search engine.^37^ These factors may allow the spread of misinformation to susceptible cohorts, as reported in a study of misinformation spread during the COVID-19 pandemic.^38^ Prioritizing the understanding of and designing solutions for such health misinformation in the age of an *infodemic* was recently highlighted as a major priority for the Office of the Surgeon General,^39^ underscoring the importance of the current study.

### Limitations

This study should be interpreted in the context of its limitations. First, spelling errors can lead to the mislabeling of discussions as having a false-positive sentiment for being associated with statins (eg, creator misspelled as crestor, or colloquialization of the phrase stating facts as statin facts) or a false-negative sentiment (eg, atorvastatin misspelled as atovrastatin). Second, user anonymity on the social media platform used in this study limits our knowledge of the demographic characteristics of the post and comment authors in this study, although social media users have been generally characterized as young in age (between 18 and 29 years),^40^ which may inform the content of discussions.

Third, statin-related content in social media discussions is visible for free to any patient looking for statin information on the internet. The data set used in this study was created from prespecified communities that were most associated with statin-related posts; thus, it may not include other statin-related posts and comments on the social media platform. While growth of statin-related content was more than 30% year on year, growth statistics on the relevant communities before 2019 were not available; thus, we could not contextualize this growth with that of the data set. Fourth, the clustering techniques used in this study may have lumped together categories that were not intuitively similar or that were not easily clinically interpretable. This limitation points to how AI approaches can assist researchers in augmenting, but not replacing, the interpretation of big streams of data.

## Conclusion

In this qualitative study, an AI method was developed to classify statin-related content on a social media platform into topics of discussion. These 100 topics were then organized into 6 thematic groups: ketogenic diets, diabetes, supplements, and statins; statin adverse effects; statin hesitancy; clinical trial appraisals; pharmaceutical industry bias and statins; and red yeast rice and statins. Such an AI approach can be used to analyze large, contemporary social media data and generate insights into public perceptions about statins. This information may help guide strategies for addressing barriers to statin use and adherence.

## Notes

Supplement 1.
eMethods.

eReferences
**eFigure 1.** AI Pipeline Overview **eFigure 2.** Histogram of Number of Discussions Per Author **eFigure 3.** Topic Evolution Over Time **eFigure 4.** Group Determination Sensitivity Analysis **eTable 1.** Topic Overview **eTable 2.** Mean Sentiment Across Subreddits

Supplement 2.
Data Sharing Statement
