# Supplementary material for: Artificial Intelligence–Enabled Analysis of Statin-Related Topics and Sentiments on Social Media
Source: JAMA Netw Open. 2023 Apr 24;6(4):e239747. doi: 10.1001/jamanetworkopen.2023.9747 (PMC10126874; doi:10.1001/jamanetworkopen.2023.9747)
Supplement: Supplement 1. — eMethods. eReferences eFigure 1. AI Pipeline Overview eFigure 2. Histogram of Number of Discussions Per Author eFigure 3. Topic Evolution Over Time eFigure 4. Group Determination Sensitivity Analysis eTable 1. Topic Overview eTable 2. Mean Sentiment Across Subreddits [file jamanetwopen-e239747-s001.pdf]

Supplemental Online Content

Somani S, van Buchem M, Sarraju A, Hernandez-Boussard T, Rodriguez F. Artificial intelligence–enabled analysis of statin-related topics and sentiments on social media. *JAMA Netw Open*. 2023;6(4):e239747. doi:10.1001/jamanetworkopen.2023.9747

eMethods.

eReferences

eFigure 1. AI Pipeline Overview

eFigure 2. Histogram of Number of Discussions Per Author

eFigure 3. Topic Evolution Over Time

eFigure 4. Group Determination Sensitivity Analysis

eTable 1. Topic Overview

eTable 2. Mean Sentiment Across Subreddits

This supplemental material has been provided by the authors to give readers additional information about their work.

© 2023 Somani S et al. *JAMA Network Open.*

eMethods

Preprocessing

To prepare the raw text scraped from Reddit for topic modeling (NLP), the following series of operations are performed. Since every post is composed of both a title and body, these two attributes are concatenated and separated by a period. Next, hyperlinks and html tags from the body of each discussion are removed to reduce content size and reduce the risk of erroneous embeddings during topic modeling. Discussions that were less than five characters in length were also removed.

Topic Modeling

Conventional topic modeling techniques, such as non-negative matrix factorization or latent Dirichlet allocation, help identify common themes or topics from a large set of documents by finding a structured representation of these documents and localizing clusters – that is, groups with common attributes. More simple methods of representing the words, such as count vectorization or term frequency-inverse document frequency vectorization, can be problematic, as they fail to consider synonyms (e.g. 'doctor' and 'physician') and context capably, thus making sentence or document level clustering tenuous. Bidirectional Encoder Representations from Transformers (BERT) models are a new, state-of-the-art technique that have generally outperformed contemporary NLP approaches in embedding words, sentences, and documents with greater contextual and semantic meaning. In this paper, a state-of-the-art topic modeling technique called BERTopic, which leverages BERT models to improve topic modeling, is used.[1]

BERTopic starts by first embedding documents using a pre-trained sentence level BERT model, Sentence-BERT, and more specifically the all-MiniLM-L6-v2 pretrained model.[2,3] It then further reduces the dimensionality of this representation using Uniform Manifold Approximation and Projection (UMAP), an unsupervised dimensionality reduction algorithm that better preserves global topology of higher-dimensional data into lower dimensions.[4] Next, it finds clusters of documents corresponding to a particular topic using HDBScan. However, this technique works best when working with extremely large datasets expected to have a medium number of clusters, with the risk of overdetection of outliers with smaller datasets. For this reason, spectral clustering, a technique that has roots in graph theory for identifying a lower-dimensional representation of what may be considered a similarity matrix between points, was chosen.[5] Since HDBScan is also capable of choosing the optimal number of clusters based on techniques in graph theory to assess for cluster stability, the standard implementation of BERTopic with HDBScan was used to calculate the optimal number of clusters for our dataset by running the model multiple times. This led to a range of numbers, which were then used to decide on the final number of clusters specified in the spectral clustering method. Once clustered, BERTopic uses a class-based term-frequency inverse-document frequency (c-TF-IDF) technique to identify keyword representations of each topic based on the documents present. Agglomerative hierarchical clustering is then performed using Ward's linkage function on the cosine distance matrix of the c-TF-IDF representations to allow for a rough hierarchical representation of topics, which can then be visually represented in a tree-based diagram.

Since the topics created by BERTopic are very granular, discovering overarching groups within these clusters became important to provide an appreciation of the overarching themes of discussion and topics in this dataset. To group these topics, UMAP and spectral clustering on the c-TF-IDF representation of each topic was performed. Since spectral clustering requires a prespecified number of clusters to be provided, a sensitivity analysis was performed by measuring the Silhouette Coefficient and Davies-Bouldin Index across a range of prespecified clusters (2 to 25) to find the optimal number of clusters.[6,7] The Silhouette Coefficient measures how the overlap and mislabeling across clusters by calculating the mean intra-cluster distance and the mean nearest-cluster distance. Values range between -1, which suggests likely misassignment of a discussion sample to an incorrect cluster, and 1, which suggests optimum assignment of a discussion point to a cluster; a value of zero indicates overlapping clusters. The Davies-Bouldin Index measures the separation of each cluster by calculating the ratio of within-cluster distances of each discussion inside a cluster to the between-cluster distances of each discussion within that cluster to that cluster's closest neighbor; lower values suggest better cluster separation and less cluster dispersion. The resulting groups were assigned a keyword representation by manually investigating the descriptors of the different topics present in that group.

© 2023 Somani S et al. *JAMA Network Open.*

Sentiment Analysis

Sentiment analysis is a form of natural language processing that classifies the sentiment of text documents into distinct categories, most commonly “positive” (“I love statins!”) or negative (“I hate statins!”).[8–10] Multiclass models have also been developed that additionally classify documents into a neutral category when these text representations may not be polarized towards a positive or negative sentiment. For example, the phrase “statins are a medication” is not opinionated and as such does not fit the mold of most traditional sentiment analysis models. Emerging techniques for sentiment analysis leverage the same transformer model architecture, with pretrained models available on open source frameworks like Huggingface.[11] To assess sentiments for each post, a pretrained BERT model, RoBERTa, trained on social media posts and freely available from the Huggingface model hub[12], was used.[13] This model is useful since it offers multiclass labels (i.e. “positive”, “neutral’, or “negative” classification of text) and has been used in recent literature investigating healthcare problems using data from social media.[14–17] The length of the input phrase was limited to 512 characters for this model. The output comprised of three probabilities assigning the likelihood that the input text would have a negative, neutral, or positive sentiment. For phrases less than or equal to 512 characters, the preprocessed text was passed with assignment of the sentiment with the highest probability to that phrase; for instance, if a phrase had the following probability array (0.1, 0.3, 0.6) for (negative, neutral, positive), then that phrase would be labeled as ‘positive’. To process longer phrases, multiple phrases from each post or comment exceeding 512 characters were created by searching for all instances of any of the search words (e.g. “statin”, “lipitor”) and taking the 256 characters before and after that match location. Sentiment value (‘positive’, ‘negative’, or ‘neutral’) for that phrase was assigned by taking the mean of the probabilities across all subsampled regions and choosing the sentiment with the highest probability. To understand how sentiments varied across topics and groups, the sentiment label was transformed from ‘negative’, ‘neutral’, and ‘positive’ to -1, 0, and 1, respectively. Average sentiment values for each topic or group were then determined by arithmetic mean of the constituent sentiments. Average sentiment values close to -1 reflect a predominantly negative sentiment, those close to 0 reflect an overall neutral sentiment, and those close to 1 reflect an overall positive sentiment.

© 2023 Somani S et al. *JAMA Network Open.*

eReferences

1. Grootendorst M. BERTopic: Neural topic modeling with a class-based TF-IDF procedure [Internet]. arXiv [cs.CL]. 2022;Available from: http://arxiv.org/abs/2203.05794

2. sentence-transformers/all-MiniLM-L6-v2 · Hugging Face [Internet]. [cited 2022 Dec 2];Available from: https://huggingface.co/sentence-transformers/all-MiniLM-L6-v2

3. Reimers N, Gurevych I. Sentence-BERT: Sentence Embeddings using Siamese BERT-Networks [Internet]. arXiv [cs.CL]. 2019;Available from: http://arxiv.org/abs/1908.10084

4. McInnes L, Healy J, Melville J. UMAP: Uniform Manifold Approximation and Projection for Dimension Reduction [Internet]. arXiv [stat.ML]. 2018;Available from: http://arxiv.org/abs/1802.03426

5. von Luxburg U. A Tutorial on Spectral Clustering [Internet]. arXiv [cs.DS]. 2007;Available from: http://arxiv.org/abs/0711.0189

6. Rousseeuw PJ. Silhouettes: A graphical aid to the interpretation and validation of cluster analysis. *J Comput Appl Math*. 1987;20:53–65.

7. Davies DL, Bouldin DW. A Cluster Separation Measure. *IEEE Trans Pattern Anal Mach Intell*. 1979;PAMI-1:224–227.

8. Wolf T, Debut L, Sanh V, Chaumond J, Delangue C, Moi A, Cistac P, Rault T, Louf R, Funtowicz M, Davison J, Shleifer S, von Platen P, Ma C, Jernite Y, Plu J, Xu C, Le Scao T, Gugger S, Drame M, Lhoest Q, Rush A. Transformers: State-of-the-art natural language processing. In: Proceedings of the 2020 Conference on Empirical Methods in Natural Language Processing: System Demonstrations. Stroudsburg, PA, USA: Association for Computational Linguistics; 2020. p. 38–45.

9. Qiu X, Sun T, Xu Y, Shao Y, Dai N, Huang X. Pre-trained models for natural language processing: A survey. *Sci China Technol Sci*. 2020;63:1872–1897.

10. Sheikhalishahi S, Miotto R, Dudley JT, Lavelli A, Rinaldi F, Osmani V. Natural language processing of clinical notes on chronic diseases: Systematic review. *JMIR Med Inform*. 2019;7:e12239.

11. Models [Internet]. [cited 2022 Sep 8];Available from: https://huggingface.co/models

12. J-hartmann/sentiment-roberta-large-english-3-classes · hugging face [Internet]. [cited 2022 Aug 25];Available from: https://huggingface.co/j-hartmann/sentiment-roberta-large-english-3-classes

13. Hartmann J, Heitmann M, Schamp C, Netzer O. The power of brand selfies. *J Mark Res*. 2021;58:1159–1177.

14. Kolluri N, Liu Y, Murthy D. COVID-19 misinformation detection: Machine-learned solutions to the infodemic. *JMIR Infodemiology*. 2022;2:e38756.

15. Noraset T, Chatrinan K, Tawichsri T, Thaipisutikul T, Tuarob S. Language-agnostic deep learning framework for automatic monitoring of population-level mental health from social networks. *J Biomed Inform*. 2022;133:104145.

16. Baker W, Colditz JB, Dobbs PD, Mai H, Visweswaran S, Zhan J, Primack BA. Classification of Twitter vaping discourse using BERTweet: Comparative deep learning study. *JMIR Med Inform*. 2022;10:e33678.

17. Anetta K, Horak A, Wojakowski W, Wita K, Jadczyk T. Deep learning analysis of Polish electronic health records for diagnosis prediction in patients with cardiovascular diseases. *J Pers Med*. 2022;12:869.

© 2023 Somani S et al. *JAMA Network Open.*

eFigure 1. AI Pipeline Overview

First, candidate subreddits that contained discussions on statins and cross-matching all discussions (posts, comments) with statin-related keywords (“Dataset Constraints” box) were identified. Next, the text of each discussion point was preprocessed to permit natural language processing (“Dataset Preparation,” blue box). Then, topic modeling to identified 100 topics (“Topic Identification,” yellow box) and 6 groups (“Group Identification,” teal box), and sentiment analysis (“Sentiment Analysis,” yellow box).

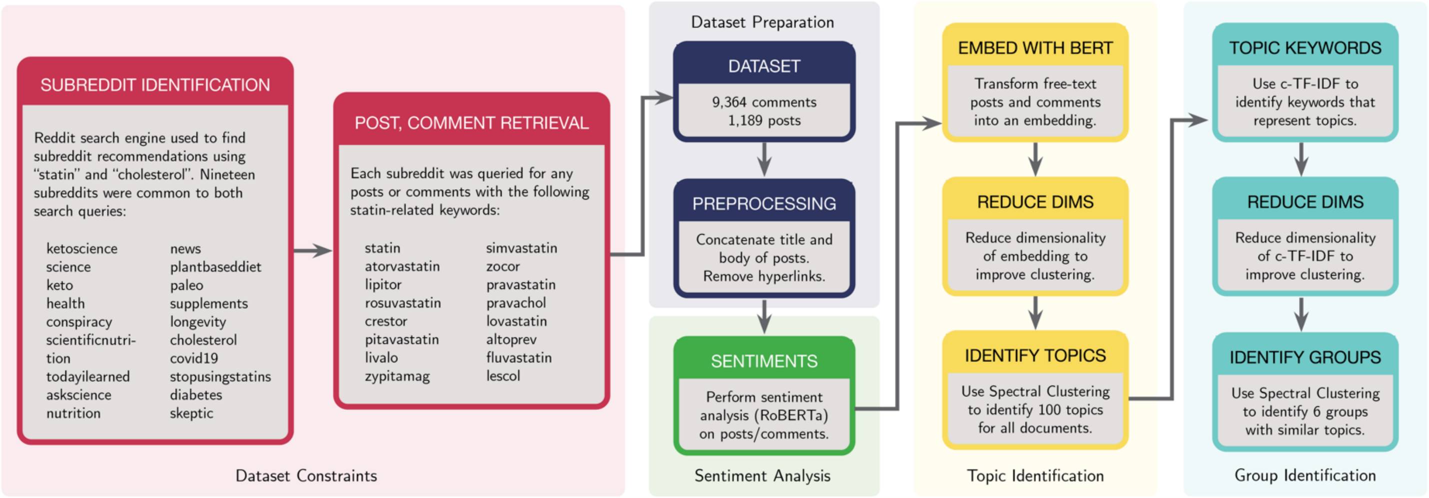

© 2023 Somani S et al. *JAMA Network Open.*

eFigure 2. Histogram of Number of Discussions Per Author

Distribution of the frequency of the number of discussions (posts and comments) made by each author. Note that a kernel-density estimator has been applied on the graph to smooth the histogram.

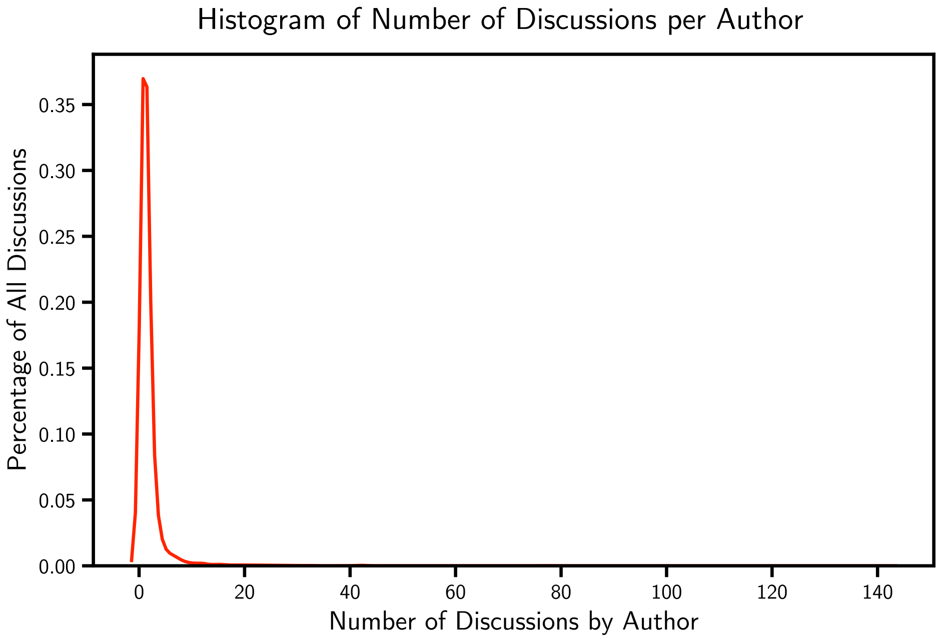

© 2023 Somani S et al. *JAMA Network Open.*

eFigure 3. Topic Evolution Over Time

Distribution of the frequency of all posts and comments contained within each topic over time.

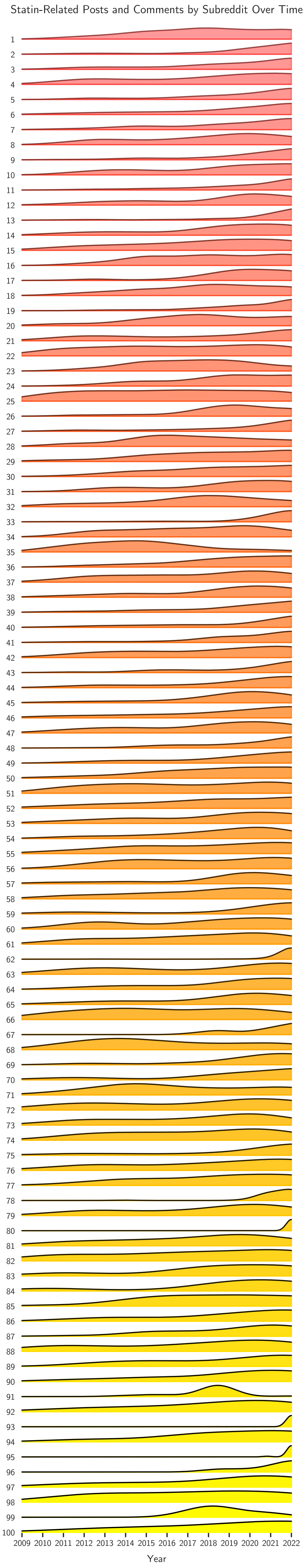

© 2023 Somani S et al. *JAMA Network Open.*

eFigure 4. Group Determination Sensitivity Analysis

Determination of the optimal number of groups for coalescing all 100 topics into by the Silhouette score (top) and Davies-Bouldin score (bottom). Higher Silhouette score and lower Davies-Bouldin scores represent better clustering performance, which was maximized at n = 6 groups (“number of clusters”).

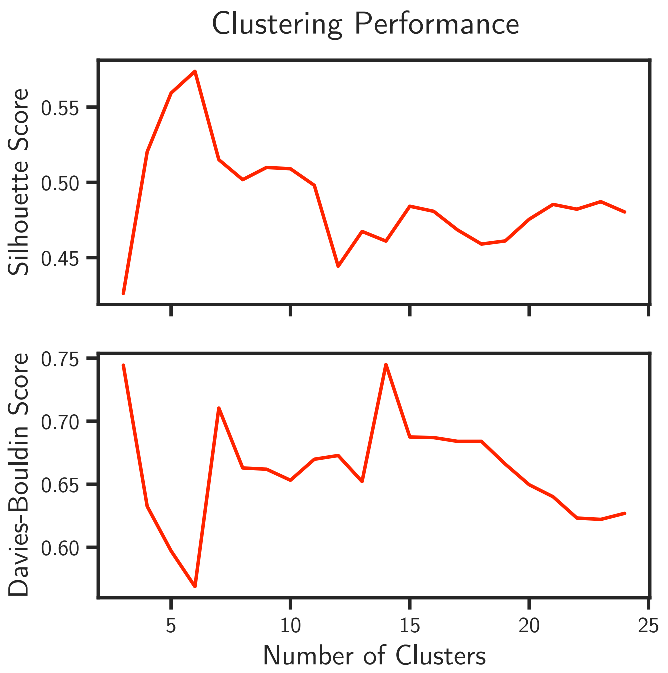

© 2023 Somani S et al. *JAMA Network Open.*

eTable 1. Topic Overview

Table on the number of discussions contained in, keyword representations for, and representative texts (text closest to the numerical mean of the cluster) of each topic. Keywords chosen are the most common words in the class-based term-frequency inverse-document frequency (c-TF-IDF) for that topic, which reflect words in that topic that were most unique to it, compared to words across other topics, during topic identification.

Topic	Posts (#)	Comments (#)	Group	Subreddit	Keywords	Representative Post
**1**	26	276	1	keto	"keto", "cholesterol", "weight", "im", "months", "numbers", "diet", "ive", "high", "results"	[NSV] Cholesterol results in......not good. Got my full lipid panel, urinalysis, thyroid, etc test results in. fasting blood sugar: 73 thyroid: normal function blood pressure: 120 over 80 total cholesterol: 264mg HDL: 54mg LDL: 199mg Triglycerides: 56mg LDL/HDL ratio: 3.7mg Chol/HDL ratio: 4.9mg While the doctor I saw was reasonably positive of keto, he was not pleased with my cholesterol at all. He is recommending I make a dietary change now so that I can lower it. If not, he's wanting me to go on a statin. For the record, I have been on keto since July 10 this year. I've lost somewhere in the neighborhood of 33lbs. While it hasn't been as dramatic as I wanted, I have lost inches quite a bit over pounds (hell, I even got my first compliment today on how good I looked). I do HIIT kickboxing 4 times a week, while I try to drink a lot of water, I have had bouts of GI trouble on and off. I get 8 hours of sleep every night, my circulation has improved, and for some odd reason, I've never been able to grow a beard all the way in, but with this diet, it has come in super thick. I champion the 73 blood sugar, 120/80 blood pressure, 54 HDL as mine was always at 28 or 30, and 56 triglycerides is astounding since I had seen them high as like 150mg once about 2 years ago. But my total cholesterol and LDL are way too high. I feel defeated and this is a terrible, terrible blow. ******UPDATE****** Nurse from the doctor yesterday just called and they want me to take a statin. I informed her I want an LDL particle test and explained briefly about inflammation and she had no clue what I was talking about and "begged" me to reconsider as the doctor recommends a statin. She told me it wasn't good "common sense" to decline a statin. I told her no and that I will re-evaluate after a particle test.

© 2023 Somani S et al. *JAMA Network Open.*

**2**	7	274	1	Cholesterol	"ldl", "high", "hdl", "cholesterol", "statin", "doctor", "risk", "heart", "numbers", "190"	So based on typical guidelines your HDL is too low. Generally you want that above 40. I saw you are taking a beta blocker and I remember reading that can actually suppress your HDL a bit. Triglycerides are above the optimal of 150 but only a little so I don’t think that would be the concern. Your LDL is high, generally anything in the 130-160 is borderline high. So the LDL wouldn’t be alarm bells bad, but probably higher than would be optimal. I think perhaps already being on six months of dieting with exercise and the number still being high might be why a Statin was recommended. However if you are still actively losing weight that can effect your cholesterol numbers a bit- as they might be slightly inflated. Overall if you don’t feel comfortable taking a Statin you can always get a second opinion. Some doctors push meds more willingly than others. For what it is worth, my LDL cholesterol was higher than yours and my cardiologist prescribed diet and lifestyle change first. If that didn’t work than we would do a low dose Statin. Still have a month to go but did a one month checkup and was able to get mine down with a better diet! Statins are very safe and so they are generally regarded as low risk drugs. The benefit will almost always put weight the risk. Just a tad off because you are so young and from my understanding statins are generally not prescribed until a bit later- unless you have some horrible numbers. (LDL over 190). But obviously I’m not a doctor. I’m just surprised they didn’t tell you to do a little diet changing first.
**3**	12	258	2	diabetes	"statin", "statins", "effects", "taking", "insurance", "im", "doctor", "dont", "good", "just"	I will never take a statin again. Do some reading of recent research on the side effects and lack of effect on morbidity or mortality.
**4**	19	240	3	Health	"statins", "statin", "effects", "cholesterol", "diet", "heart", "just", "dont", "attack", "liver"	I'm not completely anti-statin, but I'm living proof that diet alone *can* change cholesterol drastically (see post above, or below if it got downvoted). I heard what you say from one of my physicians, but every other one over the years (I've moved in and out of 4 cities in the past 20 years) has said diet can help greatly. I'm just sayin' please don't use terms like *Can't*. I've also been reading more that statins may have more of a negative effect than first believed, including an increased risk of elevated BS leading to increased risk of Diabetes II - strong enough for an FDA warning [link](There's also a risk of confusion and memory

© 2023 Somani S et al. *JAMA Network Open.*

						problems. Personally, my mother and brother had severe enough muscle pain on statins that they had to d/c its use for functional reasons.
**5**	26	219	1	Supplements	"cholesterol", "diet", "high", "statin", "exercise", "eat", "try", "fiber", "doctor", "need"	I'm not a doctor but from my understanding is CholestOff is for people who don't have genetically high cholesterol, probably won't do much for you. I have genetically high cholesterol. (450 total at the age of 15, my dad's was 600 and my uncle was 520). From my understanding of what my doctor told me was I have to stay on a statin cause my body makes it itself. Has nothing to do with my diet. Before going on a statin my doctor wanted me to try cleaning up my diet and increasing my cardio. I basically went vegan for 8 months, was running 4-10miles a day and general dedication to fitness and health. My cholesterol didn't even go down 1 point in the entire 8 months.
**6**	74	170	1	keto	"cholesterol", "statin", "doctor", "statins", "high", "numbers", "taking", "im", "just", "start"	I take a low dose statin and it been great for my cholesterol numbers.
**7**	10	232	1	keto	"ldl", "hdl", "triglycerides", "mgdl", "test", "ratio", "total", "high", "im", "diet"	Pretty much this. My LDL (250ish) and total cholesterol (325) shot up from last year to this year. But my other markers were all excellent and improved. So were my blood pressure, heart rate and several other conditions that improved from all the weight loss. Several researchers also say that a short term spike in cholesterol is common with a lot of weight loss because old cholesterol is being released from fat cells into the blood stream as the fat cells shrink. It will get processed out when weight stabilizes. This video was very eye-opening to me about the statin "studies." Anyways, I'm not a doctor, but after all my research (and I did a lot) I think LDL is pretty much irrelevant. Your HDL and Triglycerides are far more important.
**8**	61	176	3	Health	"statins", "cholesterol", "study", "heart", "statin", "benefit", "people", "disease", "drugs", "patients"	What do you think about this: ? Anecdotal evidence aside, this study shows some clear benefits of a statin. Can you show me where you are getting your recommendations that cholesterol does NOT need to be controlled in those at high risk for atherosclerosis -- MI/stroke?
**9**	5	220	1	Cholesterol	"im", "cholesterol", "high", "like", "cardiologist", "ive", "heart", "diet", "just", "family"	Hi there, I feel ya...my case is somewhat similar...it's definitely my genes that gave me a fatty liver which produces obnoxious amount of cholesterol in my body...almost everybody in my family has high cholesterol

© 2023 Somani S et al. *JAMA Network Open.*

						and large number of my family members have either died of heart related ailments or recuperating heart patients...but my current generation in the family is fairly knowledgeable and taking proper precautions....personally I exercise 1 hour daily almost 5 days a week...eat healthy and my weight has never gone above 68 KGs...I used to take Statins/ Crestor for last 2 years but my numbers never really improved...so in this lockdown I have made a decision to go off my meds...and for last one month I have felt great...in the past I have done my LDL particle size test & Die test...which showed that even with high cholesterol my chances of a blockage are very minimal...so I would suggest not to stress too much about your cholesterol readings but keep other parameters like weight, sugar & BP under control...I also plan to gets the tests done regularly just to keep tabs on the factors...of course eating healthy and staying active & away from trans-fat has it's own advantages...it will definitely improve the general quality of your life & wellbeing...all the best in your journey!
**10**	4	203	3	ScientificNutrition	"statin", "statins", "effects", "intolerance", "websites", "study", "nocebo", "people", "effect", "benefit"	Even the best statin studies show a minor effect, and of course the majority of statin trials have not published their full data sets and it is clear that there are unpublished studies. Not to mention the widespread side effects, the significant increase in risk of type II diabetes (itself a significant CV risk), and the little unpleasantness with cerivastatin, which killed 52 people and sent another 385 people to the hospital in the US. And there are unresearched [concerns about statins and cancer](I don't think that much of this is surprising given the mechanism that statins operate and the importance of cholesterol throughout the body.
**11**	4	199	2	diabetes	"statin", "doc", "im", "numbers", "doctor", "taking", "effects", "thanks", "just", "dose"	Out of curiosity did you recently start a new statin or have the dosage increased? good luck to you
**12**	14	188	4	ScientificNutrition	"mortality", "trials", "group", "risk", "prevention", "patients", "95", "ci", "primary", "placebo"	The paragraph titled "Statins Reduce Heart Attacks and Prolong Life" seems misleading. Older statin trials got these results, but modern statin trials, especially those published after 2004, have been mostly unable to lower mortality. Many of them also use questionable methodology, like no placebo. The phenomenon they're

© 2023 Somani S et al. *JAMA Network Open.*

						claiming hasn't really held for 15 years. These are some examples, but I can post the rest if requested: [10,001 patients...The risk of death from any cause also did not differ significantly between the two drug regimens (hazard ratio, 1.01; 95 percent confidence interval, 0.85 to 1.19; P=0.92)... Also used active drug in run-in period. [8888 patients...open-label...Death from any cause occurred in 374 (8.4%) in the simvastatin group and 366 (8.2%) in the atorvastatin group (HR, 0.98; 95% CI, 0.85-1.13; P = .81)... [2,410 subjects...All-cause mortality was similar...for the total cohort (5.8% atorvastatin and 5.7% placebo) and for both primary prevention (4.6 and 4.3%) and secondary prevention subjects (10.3 and 10.7%)...
**13**	16	176	1	keto	"ldl", "diet", "high", "fiber", "exercise", "statin", "changes", "try", "lifestyle", "weight"	Your LDL is super high and it's pretty concerning. Please listen to your doctor. If my patient didn't want to try another diet I would definitely start a high dose statin.
**14**	13	169	4	ScientificNutrition	"cholesterol", "levels", "disease", "ldl", "risk", "low", "heart", "tc", "blood", "patients"	LDL-C does not cause cardiovascular disease: a comprehensive review of the current literature. " ABSTRACT Introduction: For half a century, a high level of total cholesterol (TC) or low-density lipoprotein cholesterol (LDL-C) has been considered to be the major cause of atherosclerosis and cardiovascular disease (CVD), and statin treatment has been widely promoted for cardiovascular prevention. However, there is an increasing understanding that the mechanisms are more complicated and that statin treatment, in particular when used as primary prevention, is of doubtful benefit. Areas covered: The authors of three large reviews recently published by statin advocates have attempted to validate the current dogma. This article delineates the serious errors in these three reviews as well as other obvious falsifications of the cholesterol hypothesis. Expert commentary: Our search for falsifications of the cholesterol hypothesis confirms that it is unable to satisfy any of the Bradford Hill criteria for causality and that the conclusions of the authors of the three reviews are based on misleading statistics, exclusion of unsuccessful trials and by ignoring numerous contradictory observations."
**15**	37	142	3	news	"statins", "polypill", "people", "drugs", "heart", "merck",	Just do a google search for Statin studies and you will get lots of university and health official studies. About 15 years ago there was a PBS radio show called "All things

© 2023 Somani S et al. *JAMA Network Open.*

					"statin", "study", "drug", "patients"	considered" it had 10 of the countries most highly respected heart doctors on and they talked about statins. Only 1 said that he thought they were safe and could do good. The others said the risk of side effects and very little proof of benefits outweighed their wide scale usage. One even made the comment that most people who suffer heart attacks have normal cholesterol levels. Of all the doctors on the show and one of the youngest, the doctor who praised the use of statins went on to do commercials and become a spokesman for the drug. He used it for 8 years before he had his first major heart attack. The rest are all doing fine. Want to see how crazy the doctors and drug companies are in bed together? Ask all your friends how many of them are on or have been told to get on statins? Its a travesty beyond imagination.
**16**	10	161	1	keto	"keto", "ldl", "hdl", "ldlp", "high", "test", "ratio", "fat", "ketosis", "mgdl"	High LDL - 9 months of Keto. My doctor is eager for me to go back on statin medication. I stopped taking it in 2019 due to side effects. I started keto in October 2019 and am now 45lbs lighter (34yo male). In October 2019 my number were: LDL: 181 HDL: 33 Triglycerides: 332 In June 2020 my numbers are: LDL: 256 HDL: 40 Triglycerides: 107 My doctor is extremely concerned about the LDL and says it must come down. There is a lot of conflicting information out there regarding keto and cholesterol. I'm hoping to receive some guidance and sources from the gurus in the subject. Thank you.
**17**	19	132	4	ScientificNutrition	"ldlc", "ci", "95", "mortality", "events", "reduction", "cvd", "trials", "risk", "lowering"	People seem to think that statins are there to lower LDL-C. However, there are no trials to show statins work in that setting. statins don't really lower CVD events that much it's very controversial whether they lower all-cause mortality at all. These statements are not correct. [Efficacy and safety of more intensive lowering of LDL cholesterol: a meta-analysis of data from 170,000 participants in 26 randomised trials](**Across all 26 trials, all-cause mortality was reduced by 10% per 1·0 mmol/L LDL reduction** (RR 0·90, 95% CI 0·87-0·93; p<0·0001), largely reflecting significant reductions in deaths due to coronary heart disease (RR 0·80, 99% CI 0·74-0·87; p<0·0001) and other cardiac causes (RR 0·89, 99% CI 0·81-0·98; p=0·002), with no significant effect on deaths due to stroke (RR 0·96, 95% CI 0·84-1·09; p=0·5) or other vascular causes (RR 0·98, 99% CI 0·81-1·18; p=0·8).

© 2023 Somani S et al. *JAMA Network Open.*

						[Statins for the primary prevention of cardiovascular disease](**Fourteen randomised control trials (16 trial arms; 34,272 participants) were included.** Eleven trials recruited patients with specific conditions (raised lipids, diabetes, hypertension, microalbuminuria). **All-cause mortality was reduced by statins (RR 0.84, 95% CI 0.73 to 0.96) as was combined fatal and non-fatal CVD endpoints (RR 0.70, 95% CI 0.61 to 0.79).** Benefits were also seen in the reduction of revascularisation rates (RR 0.66, 95% CI 0.53 to 0.83). Total cholesterol and LDL cholesterol were reduced in all trials but there was evidence of heterogeneity of effects. There was no clear evidence of any significant harm caused by statin prescription or of effects on patient quality of life. [Efficacy of cholesterol-lowering therapy in 18 686 people with diabetes in 14 randomised trials of statins: a meta-analysis](During a mean follow-up of 4·3 years, there were 3247 major vascular events in people with diabetes. **There was a 9% proportional reduction in all-cause mortality per mmol/L reduction in LDL cholesterol in participants with diabetes (rate ratio [RR] 0·91, 99% CI 0·82–1·01; p=0·02), which was similar to the 13% reduction in those without diabetes (0·87, 0·82–0·92; p<0·0001).** This finding reflected a significant reduction in vascular mortality (0·87, 0·76–1·00; p=0·008) and no effect on non-vascular mortality (0·97, 0·82–1·16; p=0·7) in participants with diabetes. There was a significant 21% proportional reduction in major vascular events per mmol/L reduction in LDL cholesterol in people with diabetes (0·79, 0·72–0·86; p<0·0001), which was similar to the effect observed in those without diabetes (0·79, 0·76–0·82; p<0·0001). In diabetic participants there were reductions in myocardial infarction or coronary death (0·78, 0·69–0·87; p<0·0001), coronary revascularisation (0·75, 0·64–0·88; p<0·0001), and stroke (0·79, 0·67–0·93; p=0·0002). Among people with diabetes the proportional effects of statin therapy were similar irrespective of whether there was a prior history of vascular disease and irrespective of other baseline characteristics. After 5 years, 42 (95% CI 30–55) fewer people with diabetes had major vascular events per 1000 allocated statin therapy.

© 2023 Somani S et al. *JAMA Network Open.*

**18**	2	147	1	keto	"keto", "statin", "statins", "doctor", "numbers", "cholesterol", "diet", "dont", "ketone", "good"	I'm just starting out, but if Keto is my ticket to long term weight loss and my body is not self regulating cholesterol issues after a couple years, I think I would be open to a statin to help. But I plan to give my body time to figure it out. Just my two cents ATM.
**19**	39	109	1	keto	"ldl", "statin", "100", "high", "range", "doctor", "rosuvastatin", "numbers", "im", "thinks"	Nice! That means my LDL is just fine. My doctor still swears that ALL LDL is bad and wants me on a statin. I am refusing.
**20**	36	106	1	keto	"a1c", "metformin", "insulin", "glucose", "rbc", "sugar", "blood", "fasting", "diabetes", "diagnosed"	I am a 5’10” 47M SW:308/CW:273/GW:190 and have been Type II for at least 5 years. I started 8 weeks ago at 308 and today (weigh in day) saw the scale at 273 for a total loss of 35 lbs, its been several years since I saw that number. My last A1C was 10.2 just days into starting and my blood sugar measured 325 the day I started and this morning my waking blood sugar was 73 and for the last couple of weeks it has been in the mid 70’s to low 80’s. Part of me hates taking my blood reading 4 times a day (waking, prior to breaking fast, prior to dinner and two hours after dinner) but it continues to reinforce the benefits of keto and IF. I figure that once I see my doctor in April coupled with a new A1C reading that these will be reduced significantly. I am also hopeful that my medication (Metformin 3-4x500 daily, a weekly low dose Trulicity and the statin (a preventive since I have never had cholesterol issues)) will also be reduced and maybe eliminated. I track via Carb Manager and measure/weigh/log everything, I have always been very disciplined in my work life but realized I need the same discipline when it comes to my health. I adjust my macros weekly which are now 1649 calories, 20 Carbs (Net-which I am almost always under), 103 Protein (though will typically exceed but stay within my calories) and 128 Fat (which I stay under)
**21**	1	140	1	PlantBasedDiet	"diet", "eating", "eat", "paleo", "im", "oil", "foods", "like", "exercise", "cholesterol"	Now what?. So, I started a whole foods, plant based diet on April fools day (!) because I was worried for my health. My cholesterol had been high--upper 200's--for years, and I didn't want to die of a preventable heart attack. Tried a statin drug that worked very well, but caused those "stop taking immediately and contact your doctor" side effects. In May, I had blood work done; cholesterol was 243. Ok, good, going down. I've been pretty good on the plan: no meat, dairy, animal products, cooking without oil. Lots of

© 2023 Somani S et al. *JAMA Network Open.*

						beans, greens, and grains. The occasional cookie or chocolate. I lost 10 pounds. Yesterday I proudly went back to have my cholesterol checked: 247. All other blood work is normal, my blood pressure is normal/low, my BMI is fine. But my cholesterol has not budged. I'm so disappointed, and I don't know where to go from here. I'm sure, in my heart (ha), that this diet is healthier than what I was doing before, but tests are saying it really has made no difference. Do I keep going? Eat a cheeseburger? Get stricter? Sorry for the rant, but I am at such a loss.
**22**	52	86	5	news	"drug", "pfizer", "drugs", "companies", "money", "pharma", "billion", "company", "million", "research"	Man, if people only knew. In 2015, enough prescription opioids were prescribed in America to sedate every man woman and child in America around the clock for a month. That's just one drug. The whole thing is that way. Direct to public advertising has only been legal since the Reagan administration. Now, they straight up invent ailments. Research the FDA drug approval process. 2 positive studies and they control results. 49 failures and 2 good studies? It's going to market. There's even a $350k "fast lane" to rush a drug to market. If you see a prime time TV ad for a drug, there's a 99% it's bullshit. Restless Leg Syndrome? How about cutting caffeine and refined sugars? No, put em on a once a day forever pill. That'll fix it. If it causes anxiety, give me Xanax. Impotent? Here's Viagra. Uh oh, your cholesterol is high. Here's some Lipitor...and so on forever. Pharmaceutical companies are not charitable. They are corporations and they are in the business of symptom maintenance. Nothing more. No cures, only once a day pills. Doctors are not charities. They are businesses, and patients are customers. "Ask your doctor if _____ is right for you!" Why in the fuck are patients telling the doctor what drug they need? Why? Thanks for reading what I had to say, man. I really want to help people understand the problem and help steer others away if I can. I want my experience to mean something more than just a lesson for me. I wanna take the shittiness and make some good of it.
**23**	6	131	1	keto	"keto", "im", "carbs", "weight", "lbs", "blood", "ive", "pounds", "calories", "day"	I just have to brag a little. I started veeeery lazy keto in earnest on February 20th. We had a twelve week "Biggest Loser" competition at work. I went from 192.2 to 171.5! In twelve weeks! I won! I visited my doctor this week for my six month checkup. I'm on a statin for cholesterol, so I was

© 2023 Somani S et al. *JAMA Network Open.*

						interested in my bloodwork. First, I'm down exactly 30 pounds from my last visit. Fully clothed on their scale, I went from 202 to 172. The results from the bloodwork were even more incredible. My triglycerides went from 281mg/dl to 69mg/dl!!! Above all, I feel pretty great. I'm at 163 unclothed now, and I have a much better relationship with food as well as my body. For anyone thinking about it, just jump. It's easier than you think, and just plain works.
**24**		136	1	keto	"trigs", "hdl", "ratio", "ldl", "numbers", "triglycerides", "trigshdl", "statin", "good", "particle"	Whew - good for you to decline the statin. Trigs are still a bit high, and HDL seems a bit low. Are you eating enough saturated fat? That raises HDL, as does exercise. Don't worry about total cholesterol - if you are worried you have to get a particle test (I think its A1c) to see if your LDL is the good kind (large and fluffy) or the bad kind (small and dense).
**25**	9	123	5	news	"lipitor", "generic", "drug", "prescription", "drugs", "pfizer", "patent", "pharmacist", "dont", "money"	Lipitor and now off-patent and not advertised. But people really do ask their doctors for the drugs they see in commercials, even if there is a more effective and cheaper alternative.
**26**		126	4	nutrition	"ldlc", "risk", "ldl", "studies", "atherosclerosis", "cardiovascular", "apob", "disease", "association", "fh"	Both studies did not measure subtypes as I have implied. One of the studies I quoted gave a brief summary of the difference in subtypes. In short genetically defective HDL is not the same as HDL increase from diet or exercise. Its just poor extrapolation. This is a correlation explained by reverse causation, causal data shows lifelong low LDL is protective Not necessarily, this theory has been subjected to controversy and in recent publications have shown not to be true. As mentioned 17.6% of ppl with <70mg/dl LDL still had heart attacks. We have these amazing drugs that promised prevention of CVD events by lowering LDL levels to even 30mg/dl but yet the actual number of CVD events and mortality do not tally. Increased LDL was established as risk factor for heart disease back in the 60s, but LDL levels have consistently failed to accurately predict whether someone will have a heart attack or stroke. [A look at long term statin data](that included thousands of people have shown these data and its the reason why cholesterol guidelines have changed to differentiate high risk and low risk groups. To save myself time. This paper should address many of the points:

© 2023 Somani S et al. *JAMA Network Open.*

**27**	46	79	1	Cholesterol	"fish", "diet", "statin", "fiber", "exercise", "cut", "eat", "try", "months", "plant"	Cut meat and other non-veg food, sugars,sodas for few months and get checked.... I think lifestyle changes are important than taking statin (unless your levels are too high). This worked for me.
**28**	26	98	1	keto	"diabetes", "blood", "a1c", "fasting", "carbs", "type", "metformin", "keto", "diabetic", "sugar"	As both a T1 and T2 diabetic, my Diabetes Educator told me to eat 180+ grams of carbs per day and just cover it with insulin. I trusted her, but my A1C was 11+, I grew increasingly fatter, I felt like absolute garbage, and I was ALWAYS hungry. Instead of telling me to back off the carbs, she just had me do more insulin, which made me even hungrier, and had me switch to "smart" carbs like bananas, whole wheat bread, etc.. On top of that, my cholesterol numbers were absolutely horrendous, and I'm allergic to every statin on the market; my blood pressure was through the roof and I was on meds for that; and I was in stage 1 liver failure. I was also extremely depressed and had terrible anxiety because I'm also disabled with a rare genetic disorder that doesn't allow me to do much in the way of exercise and severely limits my mobility. I began Keto in April, 2019, and within \~3 months, my A1C was 5.7, my cholesterol numbers were perfect, and I'd lost \~45 pounds. My insulin use has dropped from \~150 units per day to \~10 units per day. My liver is completely normal now (though still at risk). And I've stopped taking my blood pressure meds. I still use a cane to get around, but I'm now able to walk from one end of the living room to the other, resting between "laps", as a form of mild exercise. I started at 265 pounds and I'm at 201 now, and no longer seeing that Diabetes Educator. I had a frank conversation with my PCP and told him that I don't trust the Diabetes education system because it's obviously completely full of shit and completely failed me. Even another DE I talked to temporarily (my doctor wanted me to "try one more time") recommended I consume up to 30 carbs per meal, with lots of "small" meals every day. And lots of insulin. I noped out of there as quick as I could. **tl;dr**: the decision to go with Keto as a birthday present to myself and my wife **probably saved my life**, or at least extended it a good amount. And it was really damn easy to do, even on a disability budget.
**29**	1	119	1	keto	"blood", "a1c", "im", "months", "test", "diabetes",	**Rant** Doctor wants to put me on statins, because it is a grey area if I am still a T2 Diabetic.. I got diagnosed with

© 2023 Somani S et al. *JAMA Network Open.*

					"meds", "glucose", "weight", "eating"	Diabetes almost 3 years ago with a A1C of 8.9, I also had terrible HDL and Triglycerides, but almost perfect LDL and Total Cholesterol. I went straight to Keto, and dropped to normal glucose levels in 2 weeks, and then three months later I had a normal A1C of 5.0. I also lost 75 pounds in the last few years, and now have a BMI of 21. My current A1C is 4.7. I just had more blood work done. HDL was very stubborn for the last few years, but it has finally jumped into the normal range (46 mg/dl from 28 mg/dl last year). Also Triglycerides are now at 91 mg/dl, when at diagnoses they were 162 mg/dl. I was very excited to finally see those huge improvements, but on the downside my Total Cholesterol was now 243 mg/dl and LDL of 176 mg/dl. In the last 3 months I have become super active, and started to lose 1 pound a week for the last 3 months. I have had a big increase in my appetite, and started eating a ton of cheese, red meat, and coconut oil fat bombs. It makes sense that my LDL would go up, because of sustained weight loss, and me eating a ton of cheese and other saturated fats. My doctor did not really care that I was losing weight again, or that my Trig/HDL ratio was now below 2. Or that my LDL could be explained by me using saturated fat to try to stop the weight loss, and I was going to switch to some high monounsaturated fat to play with safe with the LDL. He said that guidelines are that if you have increased LDL, and are a diabetic then you need to be on a statin. I asked if I have a normal A1C, normal glucose levels, and are on no medication, should I still be considered a diabetic when it comes to those guidelines? He then told me that is a grey area in the recommendation. On the plus side my doctor said that all he can do is say what he recommends, that it is up to me if I want to follow it. So he did not push them to hard. I have no plan to take a statin. I don't think that LDL and total cholesterol is a good indicator for heart diseas. I think research has shown that trig/HDL is the best indicator for heart diesase in a cholesterol panel. To play it safe I plan to cut out most of the cheese and replace it with more avocados, and nuts. Also I am going to try to eat fish 3 times a week.
**30**	2	115	2	keto	"statin", "taking", "youre", "gun", "looks", "want", "dont", "youd", "lol", "congratulations"	Oh, I absolutely will NOT take a statin. Thanks again for all the feedback.

© 2023 Somani S et al. *JAMA Network Open.*

**31**	19	97	3	Cholesterol	"risk", "statins", "ascvd", "statin", "effects", "people", "therapy", "guidelines", "ldl", "heart"	Like I said, not everyone gets their ldl under control with diet alone. The pharma industry literally says on their Lipitor or Crestor website that it's to be used when diet and exercise haven't worked. Diet and lifestyle changes are definitely a must, but sometimes it's not enough especially for people with a family history. Also, statins have been shown consistently to reduce your risk of cardiovascular disease and total mortality without any significant side effects. What more do you want? I'm just mad at the fact you said statins aren't healthy which is completely false in people using it for primary prevention or for diabetics. They have also been shown to literally regress atherosclerotic plaque slightly but more importantly stabilize plaques by increasing the thickness of the fibrous cap therefore reducing the risk of the plaque rupturing and causing a heart attack. they have " pleiotropic" effects too, like anti-inflammatory effects in reducing high sensitivity c reactive protein and other inflammatory responses and by improving endothelial dysfunction which is one of the first factors in the development of atherosclerosis. There is not one singular substance that targets all these factors as well as statins at the moment with very few side effects which is why it's prescribed often.
**32**	17	96	1	keto	"keto", "diets", "lowcarb", "diet", "community", "ketosis", "weight", "fat", "doctor", "ketogenic"	I'll admit, two of the three doctors did a great job of terrifying the holy shit out of me. I had to fight off nurses calling me all day leaving messages for statin prescriptions. One mouthy one told me that I could drop dead at any time, I didn't care for that attitude though and had to tell them off. I believe it was a steady combination of old school approach on cholesterol. My new doctor (he's 45 years old and an internist with ten previous years running an ER!) told me he has read lots of journals on keto. He said there is a lot of good evidence there, but there are still doubts. I argued sugar/pharma and he told me that there are probably some doctors out there who bought into it and he didn't. He did tell me he wanted balanced and a bit of a more "paleo with high fiber" approach to see if my cholesterol lowers at all that way. He indicated if it didn't then I may have a cholesterol problem that absolutely commands a statin. 3 weeks off keto and I'm down 30ish points in total and LDL already which is comforting. I see him again in a month and if I am down another 30 points then I am in a healthy range and

© 2023 Somani S et al. *JAMA Network Open.*

						that would be quite a feat. Don't get me wrong, I cold-called a dozen or more doctors offices asking if they approved of keto diets and all rejected the idea. I would be happy to share my results in about 30 days to see if I continued to lower. For all intents and purposes, I did enjoy keto as I am a fan of dairy, but I have lowered my intake of that to almost zero right now. The higher fiber has definitely normalized my GI tract.
**33**	2	109	2	science	"atorvastatin", "rosuvastatin", "muscle", "pravastatin", "effects", "taking", "pain", "im", "mg", "10mg"	Are you having side effects? I have none from 10mg atorvastatin.
**34**		110	5	news	"insurance", "price", "cost", "pay", "costs", "drug", "generic", "pharmacy", "prices", "goodrx"	The cost of drugs truly is crazy. I work in a pharmacy and look at the cash cost of a lot of things just out of curiosity. Atorvastatin (generic lipitor) at a moderate dosage is like $450/month cash price at my pharmacy. That's obviously nowhere near the most expensive medication, but it's a very common one and it's much cheaper than most other maintenance medications we sell. I returned a Humira insulin pen that had been sitting in our fridge for a couple weeks back to our supplier a couple days ago. Got a return receipt for $5400. One freaking pen!
**35**	11	98	5	news	"generic", "patent", "drug", "trademark", "companies", "company", "generics", "drugs", "lipitor", "brand"	Can someone please explain to me how this drug isn't a readily available generic drug produced by numerous different companies by now? Most drugs go off-patent in 7 years, as evidenced by Pfizer losing a shit ton of market cap when Lipitor went off-patent. If you can sell this drug for $750 a pill, how aren't there more companies competing?
**36**	1	107	1	keto	"fasting", "lipids", "triglycerides", "nonfasting", "lipid", "statin", "numbers", "hdl", "panel", "test"	Don't get on a statin. Your doctor is the type that medicates for everything out of range. Your triglycerides are low and your ratio of hdl to ldl seems good, much better than mine. When I was finally put on statins I was about ten time higher on triglycerides and take your total cholesterol but drive hdl down to less than 30 for a few years.
**37**	2	105	2	diabetes	"lipitor", "father", "doctor", "menopausal", "effects", "taking", "bring", "ambien", "im", "ive"	Thank you, that makes me feel better. My father told me horror stories about Lipitor so I've had that in the back of my mind since my doctor mentioned this possibility six months ago.
**38**	10	96	3	conspiracy	"statin", "m8", "sub", "internetdriven", "coined",	What you say is true but how many times have you seen CNN or Fox or any of the media cover the topic of statin effectiveness? How many time have you seen anyone talk

© 2023 Somani S et al. *JAMA Network Open.*

					"cult", "steven", "source", "doesnt", "phrase"	about the over prescribing of statins and their side effects? I would wager very very little. While people who seek out the information know the truth, the vast majority of the populace has not a clue to what's really going on.
**39**	4	101	2	keto	"statin", "cardiologist", "numbers", "taking", "doctor", "statins", "heart", "test", "months", "start"	Thank you for the reply. I am going to talk to the cardiologist and ask for more precise testing before I consider taking a statin.
**40**	3	100	2	keto	"crestor", "pain", "effects", "10mg", "mg", "ive", "taking", "days", "im", "havent"	Yeah, I'm on 20mg crestor daily.
**41**	4	96	3	ketoscience	"ldl", "risk", "statin", "factors", "dense", "statins", "people", "high", "low", "ldlc"	LDL has strong impact on immune is BS. The impact is very minimal. Do not discourage people from taking statin as the effect of cardiovascular disease is very real.
**42**	23	76	1	diabetes	"mg", "metformin", "antiaging", "aging", "dhea", "bipolar", "tame", "know", "supplements", "tea"	UPDATE ; Dr agreed with ditching the statin. Switched from Janumet XR which is Januvia 100/ Metformin 1000 combo to just plain Januvia 100 per my request. (I never really adjusted to the metformin but could deal with it working from home, I'm just tired of the.. side effects. Dr was quite accommodating of my medication requests, I think he is just shocked someone is getting better and has actually taken the time to research the meds, condition, diet etc.
**43**	6	93	2	Cholesterol	"repatha", "pain", "effects", "rosuvastatin", "taking", "zetia", "atorvastatin", "muscle", "im", "10mg"	Good luck, I really hope it works for you. Barring any complications it does seem that statins are very effective. I got on 10mg rosuvastatin several weeks ago, and my cholesterol dropped from 225 to 149, with LDL going from 129 to 71. I don't have any side effects either but in any case, side effects or not nobody wants to jump on lifelong medication, right? So I'm glad it's working for me at this dose. Too bad just diet and exercise did not work as well in the past, but I'm happy taking the statin now to reduce my risk.
**44**	4	92	2	conspiracy	"lipitor", "pain", "effects", "taking", "80mg", "im", "insomnia", "zetia", "crestor", "switched"	Wait what's wrong with lipitor? My doc just prescribed it to me due to an unusually high cholesterol count even though I have a very good diet and exercise regularly..
**45**	4	91	4	nutrition	"saturated", "disease", "heart", "chd", "fat", "events", "hypothesis", "intake", "coronary", "cardiovascular"	There's no simple answer to this... The short answer is that there were a lot of dubious decisions made starting in the 1970s. Some were made because of the influence of money, some because of pet theories, and some based on bad or fraudulent science. Despite overall cholesterol

© 2023 Somani S et al. *JAMA Network Open.*

						being de-emphasized there is still a large emphasis on LDL cholesterol levels in blood. This concern here aligns quite well with a hugely lucrative statin industry, which brings in about $20 billion every year. There is decent evidence that there is a fair bit of selective publication on the statin trials and much of the data is not available publicly for peer review. You can decide yourself what you think of that situation. And most organizations - AHA, ADA, etc. - are still aligned around low fat and low cholesterol diets. My personal favorite is the Center for Science in the Public Interest (CSPI), who back in the 1980s did a campaign that resulted in fast food restaurants stopping using tallow and other animal fats for deep frying. The alternative was hydrogenated oils, which CSPI asserted was more healthy. So, everybody switched. And it turned out, of course, that hydrogenated fats were just loaded with trans fats which are very bad from a heart health perspective. When this became well-known in the early 1990s, CSPI reversed course and stopped recommending trans fats. That was a good thing, though they a) pretended that they weren't the ones who pushed for more widespread use of trans fats in the first case, b) pretended they hadn't said that trans fats were fine, and c) recommended polyunsaturated oils for deep fat frying, which are a really bad choice because they oxidize easily into some pretty nasty compounds. Anyway, that is most of why we're in the state we are, and why for years the recommended diet for diabetics was a low-fat/high-carb one. If you want to learn *way* more about the history of this, I like Nina Teicholz "The big fat surprise".
**46**	10	85	3	askscience	"alzheimers", "dementia", "brain", "cognitive", "amyloid", "memory", "statins", "function", "impairment", "cholesterol"	I think the evidence is clear statins interfere with many essential cholesterol functions. there is a paper out there linking them to Alzheimers. quote from "Now a new study published in the December 1, 2004American Journal of Medicine points up another side effect of the drug simvastatin (Zocor), which is that it reduces cognitive function, in this case attention, working memory and overall mental efficiency. The same team at the University of Pittsburgh that did this study also found that statin drugs reduce blood levels of omega-3 fatty acids which are well documented to be essential for good brain function."

© 2023 Somani S et al. *JAMA Network Open.*

**47**	13	79	4	keto	"cholesterol", "fat", "fats", "saturated", "dietary", "consumption", "egg", "heart", "disease", "telomere"	Butter nonsense: the rise of the cholesterol deniers. Butter is back. Saturated fat is good for you. Cholesterol is not the cause of heart disease. Claims along these lines keep finding their way into newspapers and mainstream websites – even though they contradict decades of medical advice. There is a battle going on for our hearts and minds. According to a small group of dissident scientists, whose work usually first appears in minor medical journals, by far the greatest threat to our hearts and vascular systems comes from sugar, while saturated fat has been wrongly demonised. And because cholesterol levels don't matter, they argue, we don't need the statins that millions have been prescribed to lower them. A high-fat diet is the secret to a healthy life, they say. Enjoy your butter and other animal fats. Cheese is great. Meat is back on the menu. This is more than bad science, according to leading scientists and medical authorities. It will cost lives. "Encouraging people to eat more saturated fat is dangerous and irresponsible," is a typical verdict, in this case from Prof Louis Levy, the head of nutrition science at Public Health England (PHE). "There is good evidence that a high intake of saturated fat increases your risk of heart disease. We need to think about where the sources of saturated fat are and how we can reduce them. The largest contributions are dairy products, including butter, and meat and meat products." The advice from PHE, the World Health Organization, the British Heart Foundation (BHF), Heart UK and other institutions and top academics is consistent. Butter and cheese may be fine in modest amounts in a balanced diet, but the saturated fat that they contain is potentially risky. Too much of it causes the liver to overproduce "bad" LDL cholesterol, which is implicated in heart disease. Mainstream scientists usually keep their disquiet to themselves. But last week, some broke cover over what they see as one medical journal's support for advocates of a high-fat diet. More than 170 academics signed a letter accusing the British Journal of Sports Medicine of bias, triggered by an opinion piece that it ran in April 2017 calling for changes to the public messaging on saturated fat and heart disease. Saturated fat "does not clog the arteries", said the piece, which was not prompted by original research. "Coronary artery disease is a chronic inflammatory disease and it can be reduced effectively by

© 2023 Somani S et al. *JAMA Network Open.*

						walking 22 minutes a day and eating real food," wrote the cardiologist Aseem Malhotra and colleagues. The BHF criticised the claims as "misleading and wrong". David Nunan, from Oxford University's centre for evidence-based medicine, and three colleagues wrote a rebuttal that the journal at first did not use and then, more than a year later, put behind a paywall, while the original article was free. Last week's letter of complaint asked Dr Fiona Godlee, the editor-in-chief of the BMJ, which publishes the British Journal of Sports Medicine, to intervene, saying the journal had run 10 pieces advocating low-carb diets and criticising statins in the past three years and that the reluctance to run the rebuttal showed a bias and lack of transparency. She replied defending the journal's right to challenge "the status quo in some settings", but allowed free access to the rebuttal. Every time a new review or opinion is published in an obscure or unlikely journal – sports medicine is, after all, primarily about helping the fit get even fitter – it is picked up by newspapers that know statin scares sell. Very often in the UK they quote Malhotra, a charming and telegenic young cardiologist in private practice whose website describes him as "one of the most influential and effective campaigning doctors in the world on issues that affect obesity, heart disease and population health". He is, it says, "not just a cardiologist. This is a man who wants to change the world one meal at a time by not just rocking the system but by rebuilding it." Malhotra urges a low-carb, high-fat diet. His book, The Pioppi Diet, has the distinction of being named by the British Dietetic Association as one of the five worst "celeb" diet books in Britain – celebrities who have tried it include MPs Keith Vaz and Andy Burnham. It includes lots of fruit and vegetables, olive oil and fish, but otherwise "hijacks" the Mediterranean diet, says the BDA. "The authors may well be the only people in the history of the planet who have been to Italy and come back with a diet named after an Italian village that excludes pasta, rice and bread – but includes coconuts – perhaps because they have a low-carb agenda," says the BDA. "The suggestion that this Italian village should be associated with recipes for cauliflower-base pizza and rice substitute made from grated cauliflower or anything made using coconut oil is ridiculous. It also uses potentially dangerous expressions like 'clean meat' and encourages

© 2023 Somani S et al. *JAMA Network Open.*

						people to starve themselves for 24 hours at a time every week." Malhotra was appointed as the first medical director of Action on Sugar, formed in 2014 by Graham MacGregor, a professor of cardiovascular medicine. Two years later, the group agreed to go their separate ways. By that time, Malhotra was expressing strong views about statins, claiming in a BMJ article that was later partially retracted that they caused side-effects in 20% of patients. On BBC radio, he went further. "It was actually probably an underestimate," he said, and questioned the benefits of the drug for any patient, citing the cholesterol sceptic Michel de Lorgeril. He was accused by Prof Rory Collins at Oxford University of endangering lives. Collins said scare stories about statins could do as much harm as Andrew Wakefield did when he claimed that vaccines caused autism. When it comes to statins, there is a huge database of research. Since 1994, the Nuffield department of population health at Oxford University, led by two eminent epidemiologists, Collins and Prof Richard Peto, has been amassing and analysing the data in order to figure out how well they work in preventing heart attacks and strokes. They have published many papers. In 2016, in a major review in the Lancet, they concluded that lowering cholesterol over five years with a cheap daily statin would prevent 1,000 heart attacks, strokes and coronary artery bypasses among 10,000 people who had already had one. It would also prevent 500 in people who were at increased risk, for instance because of high blood pressure or diabetes. "Our review shows that the numbers of people who avoid heart attacks and strokes by taking statin therapy are very much larger than the numbers who have side-effects with it," Collins said at the time. Most side-effects can be reversed by stopping the statin, he pointed out – but heart attacks cause permanent damage. "Consequently," he said, "there is a serious cost to public health from making misleading claims about high side-effect rates that inappropriately dissuade people from taking statin therapy despite the proven benefits." But the cholesterol sceptics and statins critics reject the evidence on the basis that the trial data is from big pharma and that the raw data is not in the public domain. Maryanne Demasi, a journalist in Australia whose TV programmes questioning statins were pulled from the ABC network because of concerns over impartiality, wrote

© 2023 Somani S et al. *JAMA Network Open.*

						in January – again in the British Journal of Sports Medicine – of a "crisis of confidence" in the public because "the raw data on the efficacy and safety of statins are being kept secret and have not been subjected to scrutiny by other scientists … Doctors and patients are being misled." There were cholesterol sceptics before statins existed, doubting the hypothesis that high cholesterol in the blood, particularly in the form of LDL, furs up the arteries, leading in the worst cases to a blood clot that can trigger a heart attack or stroke. Yet, says Dermot Neely, a consultant in clinical biochemistry and metabolic medicine and a founder trustee of the Heart UK charity: "The cholesterol hypothesis is supported by a vast amount of scientific data." Recently, an expert paper was published by the European Atherosclerosis Society summarising all the evidence, to try to silence the sceptics. But they won't be silenced. A website called Thincs – The International Network of Cholesterol Skeptics – links to published and unpublished papers as well as the various books its members have written, including a joint one entitled Fat and Cholesterol Don't Cause Heart Attacks. And Statins Are Not the Solution." The director and author of many dissident papers is Uffe Ravnskov, a Danish doctor living in Sweden who has been an independent researcher, not part of any university, since 1979. His most recent review, with 15 others who are mostly members of Thincs, was published last month in the Expert Review of Clinical Pharmacology – an obscure source for newspaper stories that has been brought to the attention of media in the US and the UK, including the Daily Express, which has run many anti-statins pieces. "There is no evidence that high levels of 'bad' cholesterol cause heart disease and the widespread use of statins is 'of doubtful benefit', according to a study by 17 [sic] international physicians," said the newspaper. That is flat-earthism, says Collins. "The claims that blood LDL cholesterol levels are not causally related to cardiovascular disease (which is really in the same realm as claiming that smoking does not cause cancer) are factually false," he maintains. He believes there is an argument for refusing to give cholesterol-deniers a platform, just as some will no longer debate with climate change sceptics. Neely says a lot of people ring the nurses and dieticians staffing the Heart UK helpline after

© 2023 Somani S et al. *JAMA Network Open.*

						reading such stories or hearing about them from family and friends. “We’re very concerned whenever these messages result in people stopping a statin that they were prescribed after their heart attack. Every time there has been a statin scare story in the papers, there is a wave of people who just stop picking up their prescriptions. And as a result of that, many will probably be readmitted with another heart attack down the line,” he says. Some of those are young people who have high cholesterol from birth because of a mutated gene. One of Neely’s patients is a young man whose grandfather and father died of heart attacks at 50. He is on a statin and will be the first in three generations to escape that fate, says Neely. Asked how he can be sure of his position when the vast majority of top research scientists disagree, Ravnskov says: “Because I am right. The reason why the so-called experts say that I am mistaken is that the vast majority are paid generously by the drug companies.” Asked to elaborate, since statins are out of patent and therefore no longer make money for the companies that originally put them on the market, he expounds on the corruption, illegal practices and wealth of pharmaceutical companies. The Oxford researchers, including Collins, have published their funding. The unit has research funds from pharmaceutical companies, but the individuals do not take money from them. Ironically, say the researchers, if people refuse statins because of concerns over side-effects, they may be put on expensive newer drugs to lower their cholesterol – and this will make money for big pharma. A furore was triggered by the recommendation by the National Institute for Health and Care Excellence in 2014 that millions more people should be offered statins. Anybody who has a 10% chance of a heart attack in the next 10 years – judged on factors including weight, age and blood pressure – should consider taking a statin, it said. Anybody who has already had a heart attack or stroke is strongly advised to take one. Because the patents had expired, the pills had become highly cost-effective. That means statins are given to healthy people to prevent disease and side-effects have become a major issue. The stories are so widespread that people repeat them as if they are incontrovertible, yet the evidence from trials is that even the much-discussed muscle pain is rare. The sceptics dismiss that evidence.

© 2023 Somani S et al. *JAMA Network Open.*

						Those trials were funded by big pharma, they say, which had a vested interest in hiding any problems with the drugs. Some side-effects may be caused by interactions with other drugs people are on, such as antibiotics. But there is also evidence that some people get muscle pain because they expect to after everything they have heard. It is called the nocebo effect. The dissidents' arguments are attractively simple. Eat fat, avoid carbs and don't take the tablets, says Malhotra – who declined to answer questions for this article. We would probably all agree that we should ditch junk food and eat well instead of taking pills. But, realistically, telling people to "eat good food" isn't going to cut it. The majority of people in the UK and the US are now overweight or obese, with all the heart and vascular problems that brings, and the trend is ever upwards. One thing is for sure – the dissidents are not going to shut up shop. "My belief about the cholesterol sceptics is that they are a bit like religious fundamentalists," said Neely. "They are not open to argument. Whatever argument you present, they will find another argument because this basically defines who they are." He cites a cardiologist in the 1980s, Prof Michael Oliver, who was a sceptic of the cholesterol hypothesis that more LDL increased the risk of heart attacks and strokes. Oliver did a U-turn as more evidence accumulated, saying: "When the facts change, I change my mind." But, says Neely, "unfortunately the cholesterol sceptics we know currently don't do that".
**48**	1	91	2	Cholesterol	"pain", "muscle", "joint", "statin", "statins", "coq10", "taking", "fog", "cramps", "dose"	That's very consistent with statins. Top 3 adverse events with the class are muscle aches (myalgia), joint aches (arthlagia), and fatigue. Call your doctor and see what they say. Typically, the recommendation is to take a 2-week holiday and then try a different statin at a lower dose.
**49**	19	69	3	Supplements	"coq10", "q10", "energy", "fatigue", "coenzyme", "statin", "supplementation", "supplements", "ubiquinol", "taking"	Statins will deplete CoQ10 in your body. If you are on a statin, you may want to take it continuously.
**50**	3	83	1	diabetes	"insulin", "modafinil", "sugar", "blood", "cgm", "bg", "metformin", "diabetes", "sugars", "im"	Being put on drugs just because it's recommended has never sat well with me. I was put on a statin several years ago because my cholesterol, while still within the normal range for a regular human, wasn't low enough for a diabetic. So I did the good patient thing and started taking

© 2023 Somani S et al. *JAMA Network Open.*

						the statins (even though I was in my lower 30s at the time.) Turns out it made my dawn phenomenon worse. Not sure if it's my low-carb diet, my physiology, or what, but when I stopped taking it, my sugars came back down. My endo was on-board with my decision after seeing the data I provided. TLDR; Follow your doctor's advice, but don't be afraid to challenge them if you aren't feeling well, have negative reactions, and have some good documentation to back yourself up.
**51**	15	67	5	science	"drug", "medicine", "drugs", "pharma", "patients", "cost", "doctors", "research", "medical", "people"	Conversely: *Modern medicine* "is fine as long as people practicing it don't disregard" *conventional medicine* and acknowledge the limitations of *modern* methods. To be clear, I'm not advocating for alternative medicine here, I just want to point out that modern medicine has some very real, serious flaws and, at least in the United States, most all of them can be traced to the financial interests of pharmaceutical companies and HMOs. (Google "FDA revolving door".) Since deaths by pharmaceuticals now outpace automobile-related deaths in the US, and hospital-related infections kill nearly 100,000 people a year, it seems that the biggest problem in medicine today isn't some jackass selling a homeopathic for 4 bucks at the local Walgreens. Look at the recent spate of articles admitting that neuroscientists have no idea whether [SSRIs](work after doctors have been prescribing them on a wide scale for two decades. Or how about such mega-hits as Vioxx, Phen-fen, Thalidomide, Avandia, Acetomenophin (liver failure), Meridia, Avastin and Zocor. Phen-fen was on the market for 24 years before the FDA pulled it. In 2008 alone, Genentech sold about 2.6 **billion** dollars worth of Avastin, a breast cancer drug that has since been proven ineffective, after 5 years on the market and nearly 10 billion in sales. In contrast, in 2009, the entirety of homeopathic sales in the United States didn't even reach a billion dollars. I'm not saying that so-called "alternative medicine" should be handled one way or the other, but it is pretty clear that "modern medicine" has significant and grave problems that should be higher priority than poking at herbal remedies and homeopathics.
**52**	1	81	1	keto	"diabetes", "diabetics", "doctor", "cholesterol", "im",	I suggest you look into the horrors of statins before you ever consider letting a DR talk you into 'fixing' your cholesterol. They have a 40% chance to give you

© 2023 Somani S et al. *JAMA Network Open.*

					"diabetic", "statin", "statins", "said", "t2"	diabetes. Yeah, that's the percentage of people that WILL GET diabetes on statins, that otherwise wouldn't. Horrific, right? They also cause muscle pains--for life--as a side effect in many people. They strip coq10 out of your nervous system and cause massive problems. Drs around the world are taking on guidelines to *not* give people over 70 Statins--no matter what their cholesterol numbers are, because they've been proven to reduce quality of life, increase arthritis, and create complications leading to premature death. If it's not good for them, why is it good for anyone else? And, they do not--in ANY study, lower the risk of stroke or heart attack on people who have NOT already had one. They're not proven to extend lifespans (only about 3%)--mostly because that diabetes thing kills you faster. So, unless you've already had a heart attack or stroke, dont even consider a statin unless you, yourself, dig into some of the research. Some other people in the comments posted a few links, and you can start there. The one shows how they've missed the mark on health cholesterol levels by several HUNDRED points, and that *lower* cholesterol has a *higher* association with premature death than higher does. No matter what my cholesterol numbers are, i know for a fact i'm not going to play with statins. But, you'll have to look into it and decide for yourself.
**53**	3	78	1	Cholesterol	"doctor", "doctors", "statin", "cholesterol", "high", "diet", "want", "im", "hes", "said"	Doctor wants him on statin and said no amount of diet and exercise and will lower his cholesterol levels. What do you guys think?
**54**	6	73	4	nutrition	"saturated", "fat", "polyunsaturated", "fats", "cvd", "intake", "dietary", "unsaturated", "trials", "replacement"	Instead of looking at correlations look at the actual studies “ Abstract Cardiovascular disease (CVD) is the leading global cause of death, accounting for 17.3 million deaths per year. Preventive treatment that reduces CVD by even a small percentage can substantially reduce, nationally and globally, the number of people who develop CVD and the costs of caring for them. This American Heart Association presidential advisory on dietary fats and CVD reviews and discusses the scientific evidence, including the most recent studies, on the effects of dietary saturated fat intake and its replacement by other types of fats and carbohydrates on CVD. In summary, randomized controlled trials that lowered intake of dietary saturated fat and replaced it with polyunsaturated vegetable oil reduced CVD by ≈30%,

© 2023 Somani S et al. *JAMA Network Open.*

						similar to the reduction achieved by statin treatment. Prospective observational studies in many populations showed that lower intake of saturated fat coupled with higher intake of polyunsaturated and monounsaturated fat is associated with lower rates of CVD and of other major causes of death and all-cause mortality. In contrast, replacement of saturated fat with mostly refined carbohydrates and sugars is not associated with lower rates of CVD and did not reduce CVD in clinical trials. Replacement of saturated with unsaturated fats lowers low-density lipoprotein cholesterol, a cause of atherosclerosis, linking biological evidence with incidence of CVD in populations and in clinical trials. Taking into consideration the totality of the scientific evidence, satisfying rigorous criteria for causality, we conclude strongly that lowering intake of saturated fat and replacing it with unsaturated fats, especially polyunsaturated fats, will lower the incidence of CVD. This recommended shift from saturated to unsaturated fats should occur simultaneously in an overall healthful dietary pattern such as DASH (Dietary Approaches to Stop Hypertension) or the Mediterranean diet as emphasized by the 2013 American Heart Association/American College of Cardiology lifestyle guidelines and the 2015 to 2020 Dietary Guidelines for Americans." "
**55**	9	70	4	diabetes	"ldl", "cholesterol", "levels", "hdl", "high", "tg", "risk", "elevated", "total", "low"	I will admit my bias up front. I will never again take a statin. Do some reading of current research. Discard any papers funded by statin manufacturers. A few things stand out: * High LDL is not necessarily a problem; the important factor in LDL is the proportion of harmful "dense" particles. If it is mostly "fluffy" particles then high LDL is not only not a problem it is an essential component of our bloodstream. * High triglycerides combined with low HDL is an indication that there are too many dense particles in the LDL * Low HDL is a much more important indicator of future cardiac health; the minimum target for males is 40mg/dl and for females is 50mg/dl; higher is better. * Ingested cholesterol (eggs, seafoods etc) has almost no effect on serum cholesterol. As a lateral issue for those of us using diet and exercise to aid our BG control, low carb helps reduce triglycerides and exercise helps increase HDL. I wrote a bit more on the subject with some supporting cites here: [Cholesterol, Fats, Carbs, Statins and Exercise](That was

© 2023 Somani S et al. *JAMA Network Open.*

						written a decade ago, you will find more papers on the subject since if you search.
**56**	2	75	3	ScientificNutrition	"muscle", "mitochondrial", "coenzyme", "q10", "statins", "myopathy", "adverse", "effects", "gilz", "x000d"	Station side effects are rare and greatly overshadowed by their benefits. Side effects of statins are easily reversible whereas the cardiac events prevented by statins typically have irreversible side effects. " The only adverse events shown definitely to be caused by statin therapy—ie, are adverse effects of statins—are myopathy (specifically defined as muscle pain or weakness combined with large increases in blood concentrations of creatine kinase) and diabetes, although it is likely that the risk of haemorrhagic stroke isalsoincreased.Typically,treatmentof10000patients for 5 years with an effective statin regimen (eg, atorvastatin 40 mg daily) would be expected to cause about 5 extra cases of myopathy (one of which might progress to rhabdomyolysis), 50–100 cases of diabetes, and 5–10 haemorrhagic strokes. Statin therapy may also cause symptomatic adverse events (eg, muscle pain or weakness) in up to 50–100 patients per 10000 treated for 5 years. The absolute excesses of adverse events with statin therapy are increased in certain circumstances (eg, with higher statin doses and in combination with certain drugs, or in particular types of patient or population), but they are still small by comparison with the beneficial effects. Moreover, any adverse impact on major vascular events that is caused by the excesses of diabetes and haemorrhagic stroke has already been taken into account in the estimates of the overall benefits. Even so, because statins are taken by so many people, substantial numbers of people will still experience adverse effects of statin therapy. For example, about 100 cases of myopathy would be caused each year among each million people who are prescribed statin therapy. However, whereas these adverse events are readily attributed to the statin (along with many other events that are not causally related293), it is not possible to identify those individuals in whom statin therapy has prevented a heart attack or stroke, even though these absolute benefits are much larger. For example, among each million patients taking statins for secondary prevention, about 20000 people would avoid major vascular events each year that statin therapy continues.32 In addition, whereas many of the adverse effects (such as myopathy) can be reversed with no

© 2023 Somani S et al. *JAMA Network Open.*

						residual effects by stopping the statin therapy, the effects of a heart attack or stroke are often irreversible.”
**57**	1	73	4	ScientificNutrition	"trials", "studies", "2005", "mortality", "data", "trial", "results", "drug", "x000d", "evidence"	It's not just one study... Nevertheless, if you want to assume that all studies published after the new rules can't tell us anything about mortality, then it means the statin companies haven't validated their results in the face of a rather significant integrity challenge. They've essentially been accused of cheating, along with other drug companies. We can see that, in general, large drug trials have become much less likely to get "good" results after 2000. We can also see the statin companies using "unfair" tactics, like using the active drug in the run-in period to filter out bad reactions before they could be counted in the randomized portion. They obviously have incentive to get a certain result. Given this, cheating doesn't seem unrealistic. If none of the trials published recently have been powered to detect mortality changes, it means the statin companies aren't even trying to validate their claims. Despite that, you're willing to assume everything they said before is totally true and the new publishing rules are arbitrary.
**58**	1	70	1	conspiracy	"doctors", "patient", "doctor", "prescription", "pravachol", "patients", "hes", "office", "wife", "statin"	It's true. My doctor is a statin pimp. I had high cholesterol, not dangerous high, but just considered high. He frontloaded me with statins but I refused. Told him I'd try the old diet and exercise. He got pissy about me not taking statins from the "in house" pharmacy. Six weeks later, I lowered my cholesterol damn near 70 points without any medication. He was still in disbelief. So I buy that the military industrial pharmacy complex frontloads pills on doctors offices and wants them to pitch them on the unsuspecting.
**59**		69	2	Cholesterol	"crestor", "40mg", "dose", "numbers", "mg", "taking", "diet", "total", "cholesterol", "5mg"	I’m pretty young, 24, so I don’t know if it works better or quicker for me, but after taking Crestor for two weeks, my total cholesterol went down from 313 to 197. Combined with moderate exercise and a healthy diet of course. Had been trying the healthy diet for a few months beforehand. Also on accutane which might have affected these numbers a bit, as my doctor lowered that dosage at the same time. So I’m not exactly a perfect reference.
**60**	3	63	2	keto	"nation", "facts", "statin", "island", "documentary",	Can you elaborate on your statin comment please? Thanks!

© 2023 Somani S et al. *JAMA Network Open.*

					"watch", "just", "desktop", "rhelperbot", "bug"	
**61**	1	64	4	nutrition	"study", "greger", "plantbased", "diet", "claims", "saffron", "consuming", "dairy", "shows", "vegetarians"	Here's a list of some of Greger's misleading/false claims in just [**one** nutritionfacts.org video](1. Greger claims "clinical studies have shown that a plant-based diet of primarily whole grains, fruits, vegetables, and legumes can **completely prevent heart attacks**". The **uncontolled** [study](he references looks at a handful of patients who had heart disease over a short period of time who were fed a low-fat diet... **and statin drugs!!!** Also note the diet included dairy, which Greger regularly claims is terrible for you. You can read more about just how bad this study was [here]((under the *Caldwell Esselstyn* heading). He also ignores studies such as [this one](which shows that consuming dairy and egg actually reduces heart disease risk compared to a vegan diet and concludes "there were **no significant differences** between vegetarians and nonvegetarians in mortality from cerebrovascular disease, stomach cancer, colorectal cancer, lung cancer, breast cancer, prostate cancer, or all other causes combined." Here's [one more study](he neglects to mention, showing no difference in mortality between vegetarians and non-vegetarians. 2. Greger claims that a plant-based diet can prevent 75% of cancers, using [this study](as a reference. However, what the study actually says is that 30% of cancers are caused by smoking, and only 20-42% of cancers may be preventable by diet (and, no, it doesn't say what kind of diet). 3. He cites [this study](to claim that animal fat can "paralyze and cripple arteries". This is another **uncontrolled** study of only 10 participants. It measures flow-dependant vasoactivity, which slightly slowed after the meal. Of course, interpreting slightly slowed bloodflow as "crippled arteries" is massively hyperbolic. And, with no control group, we have no idea if a vegan meal may cause the exact same effect. There is also no conclusion drawn by the study about what clinical significance the results actually have. 4. He claims COPD "can be prevented and even treated with a plant-based diet" using [this study](which used exhaled NO as a measure of inflammation, which indicates airway inflammation. The study shows that the levels of exhaled NO increase after a high fat meal. However, the study specifically shows **no association** between airway

© 2023 Somani S et al. *JAMA Network Open.*

						inflammation and systemic inflammation (which is associated with COPD). The study itself actually doesn't mention COPD at all, and also doesn't suggest anything about the cause of the airway inflammation being animal fat. 5. He says "we've known for 20 years that those who eat meat are 2-3 times more likely to become demented as vegetarians." This is based on [this 27-year-old study](sponsored by the Adventist church, who practice vegetarianism. The study shows the Adventists had a lower rate of dementia than non-Adventists, but keep in mind that Adventists have a relatively small gene pool which may naturally have lower rates of dementia than the general population. They also are much more active (they do manual labor their entire life) than the general population, and we know exercise helps reduce dementia risk. Unsurprisingly, **no** study on the general population has ever produced results showing vegetarianism reduces the risk of dementia. In fact, **studies have shown the opposite**, like [this one](which shows specifically that fish consumption reduces the risk of dementia. Greger conveniently fails to mention these studies. 6. He goes on to claim that Alzheimer's can be treated with a plant-based diet, citing [this study](which compares the use of a saffron extract to a drug which is known to only have a very small clinical benefit (as shown by [this study](The study concludes that there is "*preliminary* evidence of a *possible* therapeutic effect of saffron extract." It's very disingenuous to claim that the effect has been proven, and it's from an extract - the level of active ingredients are much, much higher than you could get from simply consuming saffron. And, again, saffron extract is being studied... Yes, saffron is a plant, but consuming a specific herbal extract is **not** the same as consuming a plant-based diet. 7. He claims that kidney disease can be prevented and treated with a plant-based diet based on [this study](which does shows that decreasing consumption of red meat can prevent kidney function decline. What he **neglects to mention** is that the same study shows a similar impact from consuming dairy, which doesn't fit his narrative that dairy is evil. 8. He claims that diabetes can be **cured** by a plant-based diet, despite the fact that the medical community [has established](that diabetes **cannot be cured**. You can read more

© 2023 Somani S et al. *JAMA Network Open.*

						debunking of Greger [here](by Dr. Harriet Hall. Also check out [MediaBiasFactCheck.org](which grades nutritionfacts.org as a ***CONSPIRACY-PSEUDOSCIENCE*** source, stating: "While eating a diet high in fruits and vegetables and low in red meats is scientifically proven to be beneficial, Dr. Greger takes it to a higher level promoting a 100% plant based diet. Dr. Greger and NutritionFacts.org make 'zealot' like claims about the benefits of a vegan plant based diet. NutritionFacts cherry picks information that will always favor veganism."
**62**	5	59	2	PlantBasedDiet	"1000", "sublingually", "mcg", "maintenance", "daily", "vitamin", "recommended", "dropper", "d3", "capsules"	I did hear back. I'm gonna follow their recommendation. Doctor recommended Vitamin D3 10000 units daily for the next 12 weeks follow by 1000 daily maintenance. LDL cholesterol to be below 130 recommended small amount of statin or natural red yeast rice 2 capsules twice a day. Vitamin B12 daily maintenance lifetime of 1000 mcg sublingually and you can get a dropper at the pharmacy in 1 drop sublingually would be 1000 mcg.
**63**	3	60	3	skeptic	"vaccine", "vaccines", "immune", "aluminum", "vaccinated", "covid", "zinc", "flu", "tomljenovic", "hpv"	Follow the money, eh? Once a vaccine is manufactured, it hardly rewards the pharmaceutical company for the effort: Vaccines are dramatically less profitable than other drugs. In March, Science reported that GlaxoSmithKline, the world's largest vaccine manufacturer, enjoyed worldwide vaccine sales of $4.3 billion. Contrast that figure with the profits of one drug, the cholesterol-lowering Lipitor, which grosses $6 billion a year. According to IMS Health, a pharmaceutical intelligence service, vaccines comprise only 1 to 2 percent of global pharmaceutical sales. Vaccine experts blame the low profit margins on a number of factors. For starters, the American public has come to see vaccines as something of an entitlement. Notes Neal Halsey, MD, director of the School's Institute for Vaccine Safety and professor in International Health, "We have the psychology that vaccines are very cheap." As a society, he says, we don't want to pay a fair price for prevention unless we detect a direct threat. Most diseases currently vaccinated against—the annual winter flu or childhood diseases such as measles, mumps, and rubella—either seem old-fashioned and unlikely to occur on an epidemic level, or strike the public as inconveniences that are easily survived. Many critics within industry, however, lay the

© 2023 Somani S et al. *JAMA Network Open.*

						biggest share of blame at the feet of the U.S. government, which is America's primary purchaser of vaccines—the result of Congress's 1993 creation of the Vaccines for Children program. Under this program, which was established to improve vaccination rates among the poor, the government purchases more than half of the childhood vaccines used in the United States. Such purchasing capacity gives the federal government extraordinary negotiating power over price. The government asks for—and gets—deep discounts on many vaccines. Vaccine makers and their lobbyists argue that governmental price caps act as a big disincentive to producing vaccines that historically have been high-volume, low-profit items anyway. (Unlike antidepressants or cholesterol-lowering medicines, which are taken for years and have an ever-expanding target population, vaccines are administered on a rigid schedule and for only a limited number of doses.) Consider: In 1987, the CDC paid about $11 for a dose of MMR; today the price is only $15.50.
**64**	2	61	3	diabetes	"diabetes", "insulin", "resistance", "statins", "sensitivity", "glucagon", "statin", "risk", "suppression", "nod"	A new study indicates risk for diabetes with statin use.
**65**		62	4	nutrition	"vegan", "cholesterol", "diet", "length", "telomere", "fiber", "bile", "ampx200b", "saturated", "foods"	Diet has a huge impact on cholesterol for most people, but it's hard to tease out which dietary or lifestyle components are the largest causal factors. When I went vegan I went from a high cholesterol, high saturated fat, low fiber diet to the opposite of that. I also lost about 40 lbs of weight in the process. These factors took my cholesterol from 220 to 96. Obviously this isn't medical advice, but I would definitely look into dropping the statin. Recent research shows they are just about useless at actually preventing heart attacks and CVD deaths. They might lower your cholesterol but they don't treat the root causes that cause the high cholesterol in the first place (ie diet and lifestyle), so people end up having just as many heart issues as without the drugs.
**66**	7	52	2	science	"lipitor", "oreos", "scumbag", "ads", "clever", "just", "fuck", "sales", "hes", "ya"	They're all hooked on Lipitor now.

© 2023 Somani S et al. *JAMA Network Open.*

**67**	3	56	1	keto	"cac", "plaque", "score", "cta", "zero", "calcium", "coronary", "cad", "scan", "progression"	CAC is a Coronary Artery Calcium score. It directly measures heart disease in your coronary arteries. It's a pretty cheap scan that literally takes 5-10 mins. I'm your same age and had a father who had quadruple bypass heart surgery when he was 42. I've had elevated cholesterol my whole life and since going keto my cholesterol went even higher. TC around 320. LDL was 250. HDL 54. Trigs 84. I already knew I wouldn't take a statin so I preempted my doctor by saying I would never take a statin and I asked for a CAC test. He was more than willing to have that scheduled. He was also ok with me saying I wouldn't take a statin because I more than doubled my HDL from my last years test and my Trigs dropped from 350 down to 84. I would not seriously consider taking a statin without actually looking into it and doing your own research on it. Statin use IMHO is one of the great big pharma scams committed on the population. If you can get omega 3 from natural sources that would even be better. What were your other cholesterol numbers? That would tell you much more about the state of your health.
**68**	2	56	5	todayilearned	"drugs", "drug", "viagra", "morphine", "2019ncov", "protease", "barrier", "used", "zocor", "herbal"	I think theres something to be said for the top three prescribed drugs in the US. Lipitor, vicodin, xanax, respectively.
**69**	2	56	6	Cholesterol	"rice", "yeast", "red", "lovastatin", "monacolin", "ingredient", "natural", "active", "supplements", "supplement"	Red yeast rice is a statin basically, by the way.
**70**		56	3	ketoscience	"plaque", "calcium", "regression", "plaques", "artery", "calcification", "score", "coronary", "therapy", "arteries"	Evidence supports the idea that statin therapy not only reduces cholesterol levels but also changes existing plaques to make them less dangerous. As part of this process, the plaques may become more calcified—and thus, calcium score goes up. An increasing calcium score with statin therapy, therefore, may indicate treatment success, and should not be a cause for alarm. Since statins are thought to help prevent and even to help reverse coronary artery disease, this result seems paradoxical. In 2015, a study was published in the Journal of the American College of Cardiology which helps to clarify what this increase in calcium means. Investigators

© 2023 Somani S et al. *JAMA Network Open.*

						reviewed eight separate studies which had used intravascular ultrasound (IVUS, a catheter technique) to assess the size and composition of atherosclerotic plaques in patients treated with statins. They found two things. First, high-dose statin therapy tended to shrink plaques. Second, while the plaques were shrinking, their composition was changing. After statin therapy, the volume of lipid deposits within plaques diminished, and the volume of fibrotic cells and calcium increased. These changes—converting an unstable “soft” plaque to a more stable “hard” plaque—may render a plaque less prone to sudden rupture. (This postulate is consistent with the fact that statin therapy significantly reduces the risk of heart attacks in patients with coronary artery disease.)
**71**	1	55	5	todayilearned	"grapefruit", "juice", "cyp3a4", "metabolized", "enzymes", "liver", "cyp", "enzyme", "metabolism", "interact"	You might not find this, but there's a lot of statins that can be taken with grapefruit juice. Only Atorvastatin, lovastatin and simvastatin are affected since they are metabolised by the 3A4 enzyme which grapefruit juice inhibits. Rosuvastatin (Crestor) is hardly metabolised by 3A4 and there is no significant effect from grapefruit juice. It's also just as effective as lipitor. If you REALLY love grapefruit like you describe, ask about switching (your pharm can help there)
**72**	2	54	3	keto	"lipitor", "heart", "attack", "attacks", "36", "reduction", "risk", "33", "100", "disease"	Um..no.. Our bodies produce way more Cholesterol than our Diets can. Lipitor got famous because of their commercials [Like this one](, that claimed it reduces Cholesterol by 33% which was bogus. To Borrow a Quote [from This Blog](And about that 33% reduction … I’ve mentioned this before, but it bears repeating: you have to understand how that figure was calculated. Among the relatively young men with known risk factors for heart disease who took Lipitor in a clinical trial, two of every 100 had a heart attack. Among the relatively young men with known risk factors for heart disease who took a placebo, three of every 100 had a heart attack. Yes, that’s a 33% reduction, but it also means that for every 100 high-risk men who take Lipitor, we may at best be preventing one heart attack. Notice I didn’t say we may be preventing one extra death. That’s because we don’t have the figures to analyze. The pharmaceutical companies are quick to release figures showing reductions in heart attacks, but haven’t always released the total death figures. So 2 out

© 2023 Somani S et al. *JAMA Network Open.*

						100 got a heart attack on Lipitor, while 3 out of 100 got a heart attack on a sugar pill. Getting 33% out of that is just stupid. You should watch [this Journalist report on Cholesterol and Statins](and then decide for yourself. We also have an entire section on Cholesterol in our FAQ --- Hope you decide for yourself after you have examined all the data. Good luck
**73**		54	1	keto	"food", "nutrition", "industry", "fat", "science", "health", "eat", "people", "carbohydrates", "sugar"	just so curious why a medical practitioner would advise against it. Most GP's took only 1 class on nutrition in medical school. And what most of them learned 10 (or 20 or 30) years ago turns out to be wrong. The model of nutritional health from the 70s thru the early 2000s was instituted by government policy and was based on a hypothesis by a fellow name Ancel Keys called the "diet heart hypothesis" and basically prescribes a high-carb, low-saturated fat diet with lots of fruits and veggies. What has happened since the 70s? Everyone has gotten much fatter, heart disease is off the charts, T2-diabetes is rising. And what do doctors and the nutrition industry do? Blame the victims claiming that they're not following the diet and just eating junk food. Why does this happen? Market forces. The food industry makes cheap, high-profit margin food out of refined carbs and low quality "veggie" oils (seed oil, really). The doctors are constantly bombarded by pharma companies regarding the "statin" narrative for lowering cholesterol as the "standard of care". Most doctors aren't willing to deviate from the Standard of Care. And pharma companies don't want this to change. Statins are a blockbuster drug that makes them all tons of money. When most folks switch away from the standard high-carb, low-fat diet to a low-carb, high-fat (LCHF) diet, a few things happen in the blood that will confound most doctors. * Triglycerides drop * HDL rises * HbA1c drops * Blood pressure drops And not just a little, a lot! (Cholesterol and LDL stay about the same.) And it's sustainable. It doesn't change as long as you keep Keto. And this confounds most GPs, but they can't deny that these blood markers are all radically improved. You'd think this would be obvious that the Keto (or any Low-carb) diet is a sign that it is better, but they prefer to write every case off as a special n=1 case. There's so much more to say on this, but I gotta get to work. Hope this helps.

© 2023 Somani S et al. *JAMA Network Open.*

**74**	4	49	5	nutrition	"physicians", "medical", "nps", "patient", "people", "care", "school", "doctors", "patients", "corporate"	I have to agree with the person you are responding too....however I dont blame the physicians. The device and drug companies design studies to show maximal effects and the studies that show no benefit dont get published. So the physicians are left looking at and interpreting data that are highly biased. Im a physician and as Ive grown in wisdom (and common sense) I buy into very little of what these coorporations are selling us. If you want to avoid cardiovascular disease you need to eat diet consisting of unprocessed food including severely limiting processed carbohydrates. Ive applied these principles to my own life and the results have been far more drastic than taking a statin for example ever could have been. I am a big believer that we are being sold a lie. Intentionally by some, unintentionally by others. And for the record I am confident that saturated fat (from animals) is healthy!
**75**	4	48	2	Cholesterol	"crestor", "allergy", "pain", "skin", "brand", "natural", "intolerance", "vomiting", "nausea", "arthritis"	what statin are you on? I realize people are affected differently, but I'm curious if there is one that seems be more tolerated than others. I was taking the generic version of Crestor (Rosuvastatin). Ironically I actually did better with Crestor back when I was taking an antipsychotic, which is strange because those drugs elevate cholesterol even more so I ended up having to take a higher dose. I wonder if there'd be a significant difference in side effects even just lowering it by 5mg. I remember back in the day I was on 5mg and didn't seem to have issues and it was still an effective dose. For a brief period I was on 40mg because of one of the antipsychotics I first started out with. I still have been getting 40mg since it's a much better value for some odd reason; however, I have a pill cutter and have only been taking 10mg, but maybe even the 5mg could be a big difference. Either that, or I've just reached a point now that I can't tolerate the medication.
**76**	1	51	2	keto	"simvastatin", "atorvastatin", "rosuvastatin", "effects", "astaxanthin", "dose", "aspirin", "doses", "muscle", "ive"	Simvastatin: dosage uses and side effects high cholesterol drugs. [removed]
**77**	1	51	1	diabetes	"diabetes", "stem", "diabetic", "diabetics", "glucose",	I've been on a statin for years, and my blood glucose is very well controlled.

© 2023 Somani S et al. *JAMA Network Open.*

					"statin", "cell", "type", "sugar", "statins"	
**78**	1	50	4	COVID19	"covid19", "patients", "sarscov2", "severe", "inhospital", "fig", "mortality", "use", "hospitalization", "hospitalized"	Abstract: Statins are lipid-lowering therapeutics with favorable anti-inflammatory profiles and have been proposed as an adjunct therapy for COVID-19. However, statins may increase the risk of SARS-CoV-2 viral entry by inducing ACE2 expression. Here, we performed a retrospective study on 13,981 patients with COVID-19 in Hubei Province, China, among which 1,219 received statins. Based on a Cox model with time-varying exposure, as well as a mixed-effect Cox model after propensity score-matching, we found that the risk for 28-day all-cause mortality was 5.2% and 9.4% in the matched statin and non-statin groups, respectively, with a hazard ratio 0.58. These results imply the potential benefits of statin therapy in hospitalized subjects with COVID-19. Further, they give support for the completion of on-going prospective studies and randomized controlled trials involving statin treatment for COVID-19, which are needed to further validate the utility of this class of drugs to combat the mortality of this pandemic. They have an… [interesting graphical abstract](
**79**		44	5	conspiracy	"child", "children", "trafficking", "commission", "abuse", "pedophilia", "judicial", "satanic", "law", "tribunal"	All because of corporate deception for a quick profit. Plus, everyone is prescribed a statin nowadays, but that's a topic for another day.
**80**	6	38	2	news	"fetal", "tums", "tylenol", "claritin", "sudafed", "preparation", "benadryl", "bismol", "maalox", "pepto"	the concept was tested on cells Yes and a hospital has [taken the next step](for those people too. The hospital's form includes a list of 30 common medications that used fetal cell lines during research and development. The list includes acetaminophen, albuterol, aspirin, ibuprofen, Tylenol, Pepto Bismol, Tums, Lipitor, Senokot, Motrin, Maalox, Ex-Lax, Benadryl, Sudafed, Preparation H, Claritin, Prilosec, and Zoloft.
**81**		43	1	diabetes	"hes", "breast", "cancer", "eats", "dad", "survival", "soy", "father", "metformin", "type"	Sorry to ask again. Possible type 2 misdiagnosis.. Like the flair says, husband is supposedly type 2 diabetic. He was diagnosed during a terrible bout of influenza + 3-4 secondary infections that almost took his life. He had lost a pretty serious amount of weight during his sickness, and they took his a1c, they were at 16.something. We don't have a lot of money, so we do what we can to eat as little carbs as possible. He's not eating the best, but he's not

© 2023 Somani S et al. *JAMA Network Open.*

						eating terribly either. No sweets, soda, or too many refined or simple carbs. His sugars after a meal are around 20 (sometimes a bit more or less), fasting sugars in the am at about 15-17... Even after eating nothing for long periods of time, he's still high. He's taking several medications, Invokana, Sitaglipin+Metformin long release, and a statin for high LDL (but that was a problem before). It doesn't seem to help. The doc keeps telling him he's not trying enough, so he hates going back to see him. I've been worried about type 1 from the start, because of how this all started. I'm a nutritionist, and I've never had a client with such terrible diabetes weigh as little as he does (6'3, maybe 225 lbs) or have as few bad nutritional habits. He's not even 33 yet! We're just wondering how to talk to his doctor about this. Last time we tried, he was dismissed very quickly, and we'd like to know how his doc is so sure he's type 2 so we can be at peace with it. The appointments are rushed, and we want to be sure his treatment is adequate. How would you go about having this conversation? Do you guys think we're crazy to even suspect this?
**82**	15	23	5	news	"probiotics", "offlabel", "adhd", "lipitor", "microbiome", "antibiotics", "pills", "drug", "lot", "ambien"	I was specifically thinking of adverse interactions with prescription medications like Lipitor or Klonapin.
**83**	3	34	1	Supplements	"multi", "gmc", "vitamin", "k2", "magnesium", "supplements", "powder", "k1", "iu", "mcg"	Its really hard - we don't know about your diet and health. However, with the vitamen D I would investigate K2. I also don't take every thing every day - without good reason. Example: I take a statin - so I supplement with coq-10 and k2 because both studies have said that both are reduced if you take a statin. Doctor also told me to take 5000 IU of D so the K2 is a good idea for that. I occassionally take other stuff (eg magnesium, generic multivitamen but more like a couple of times a week).
**84**	1	36	4	Health	"epa", "omega3", "dha", "acid", "oil", "fish", "cardiovascular", "fatty", "folic", "reduceit"	New research from Europe found that fish oil supplements are more effective than a popular cholesterol medication at helping people with chronic heart failure. When someone has chronic heart failure, their heart becomes enlarged and fails to fill with blood to pump it through the body. In the study, almost 3,500 patients were given an prescription formula omega-3 pill on a daily basis. About the same number of patients took a placebo. The patients were

© 2023 Somani S et al. *JAMA Network Open.*

						followed for about four years. A parallel study gave one group the cholesterol drug Crestor and placebo pills to the other group. When comparing the results from both studies, researchers found that taking fish oil (omega-3) supplements are slightly more effective than the drug. Dr. Richard Bonow, Chief of Cardiology at Northwestern University Hospital in Chicago and former president of the American Heart Association, says that “it’s a small benefit, but we should always be emphasizing to patients what they can do in terms of diet that might help.” Dr. Jose Gonzalez Juanatey from the European Society of Cardiology says these new findings may give patients a new treatment and offer a change of dietary recommendations for people with chronic heart failure. Juanatey said, “this reinforces the idea that treating patients with heart failure takes more than just drugs.
**85**	3	34	2	news	"lipitor", "taking", "prescribed", "cholesterol", "gave", "amp", "high", "dad", "told", "staten"	Get on Lipitor. It's a miracle drug. My cholesterol dropped substantially after taking it.
**86**	3	34	3	todayilearned	"niacin", "internet", "available", "pmid", "nicotinamide", "nad", "mg", "pmcid", "liter", "deciliter"	But not significantly efficacious for lowering cholesterol. Niacin can actually lead to some adverse events at high doses, especially when taking with a statin. Source - I’m a pharmacy student who did a case report on a death due to statin-induced rhabdomyolysis while taking high dose niacin.
**87**	1	33	3	diabetes	"diabetes", "pcsk9", "cells", "fung", "risk", "type", "senescent", "patients", "statins", "therapy"	I think the transcription that this linked article gives is a little misleading. Consider that the people taking statins (those with elevated blood lipids) already have significant risk of developing type 2 diabetes. Lowering someone's cholesterol without altering their diet - a la relying on statin therapy, won't lower their risk of developing diabetes if they're spiking their blood sugar every time they eat.
**88**	1	29	1	Cholesterol	"breast", "cancer", "survival", "soy", "angina", "asthma", "doctors", "mg", "onco", "al"	Thank you very much for this helpful info. My dad have diabetes, hypertension, and struggled with high cholestrol, he was also a heavy drinker. Now he is almost 70 and somewhat very healthy/sturdy because he strictly watches what he eats. Never had a stroke or a cardiac arrest but he is on several different meds taken daily to have these issues under control. It sucks but I don't have much information besides that and have no idea about what exactly are his medications now. Following your link, I

© 2023 Somani S et al. *JAMA Network Open.*

						guess my levels suggest a statin treatment, do you think these results are scary for a 27 years old?
**89**	2	24	2	Cholesterol	"lipitor", "dosing", "ldl", "mg", "dropped", "went", "20", "programmed", "crestor", "gone"	My LDL went from almost190 to 70 in four weeks of taking 10 mg of Lipitor.
**90**		25	6	Supplements	"rice", "ryr", "yeast", "red", "lovastatin", "extract", "ci", "decreased", "aorta", "mi"	red yeast rice is a statin, and statins should be avoided at all costs. If you have high cholesterol and also mind your diet, you may want to check LMHR communities and the cholesterol code. statins are basically mycotoxins and deplete you if fat soluble nutrients, like coQ10, vit D, K, A and E, and in all likelihood through these depletions worsen cardiovascular health.
**91**	2	20	1	keto	"acids", "fatty", "bloodstream", "weight", "lost", "sideways", "transient", "pounds", "mgdl", "loss"	**I lost weight and my cholesterol . . . went up!** By Dr. Davis | June 5, 2012 This is a fairly common observation around these parts: *"I eliminated wheat from my diet and have limited my consumption of junk carbohydrates like corn and sugars. I lost 38 pounds over three months and I feel great. I initially lost weight rapidly, but have more recently slowed to about 1-2 pounds per week. But my doctor checked some lab values and he flipped! He said that my HDL dropped, my triglycerides went up, and my blood sugar went up 20 points! He wants me to take a statin drug and metformin for my high blood sugar. What gives?"* Easy: You are losing weight. Let me explain. When you lose weight, you are mobilizing energy stored as fat. That fat is mobilized as fatty acids and triglycerides into the bloodstream. 10 pounds lost, for instance, means the equivalent of 35,000 calories of fat released into the bloodstream. These fatty acids are not alone. They interact with the other elements in the bloodstream. In particular, this flood of fatty acids: - Block insulin–and thereby increase blood sugar. A non-diabetic can even become transiently diabetic during weight loss. - Increase triglycerides–A starting triglyceride level of, say, 120 mg/dl, can increase to 180 mg/dl during active weight loss. (Triglycerides contain fatty acids.) - Decreased HDL–Excess fatty acids and triglycerides modify HDL particles, causing their degradation and elimination. A starting HDL of 45 mg/dl can drop to 28 mg/dl, for example. - LDL measures go haywire–The conventional calculated LDL cholesterol, or even generally superior measures like

© 2023 Somani S et al. *JAMA Network Open.*

						apoprotein B or NMR LDL particle number, can go in any direction rather unpredictably: They can go up, down, or sideways. Likewise, the (miserably useless) total cholesterol value can go up, down, or sideways. - Increased blood pressure–This is likely due to the enhanced artery constriction that occurs due to increased endothelial dysfunction, i.e., dysfunction of the normal relaxation mechanisms of arteries. The key is to recognize these phenomena as nothing more than part of weight loss and the inevitable mobilization of fatty acids into the bloodstream. Accordingly, decisions should not be made based on these values, since they are transient. Your doctor will likely try to push hypertension medication, statin drugs, fibrate drugs, diabetes drugs . . . all for a transient effect. Is there a way to not experience these changes? Sure: liposuction. To my knowledge, there is no way short of extracting fat with a trocar to avoid these changes. As a practical matter, avoid having blood drawn until weight has plateaued for at least 4 weeks and these changes are allowed to reverse. Only then will you know what you have achieved in your wheat-free adventure.
**92**		21	6	Health	"yeast", "rice", "red", "lovastatin", "fda", "sp", "product", "monacolin", "naturally", "monascus"	Red yeast rice has been used in Chinese medicine for hundreds of years. The FDA regulates the rice because Pharma companies created synthetic versions of the statin lovastatin, which was first discovered naturally occurring in the rice, and patented it. For example Mevacor, a brand-name statin, was approved by the FDA in 1987 and now available as a drug. The FDA and Pharma's attempt at patenting red yeast rice failed because it is a natural product. Since the chemical lovastatin is subjected to regulation as a drug, the rice is banned. Luckily, the FDA does not regulate dietary supplements.
**93**		20	2	news	"exlax", "motrin", "sudafed", "benadryl", "tums", "preparation", "tylenol", "ibuprofen", "senokot", "pepto"	Here you go. “The list includes Tylenol, Pepto Bismol, aspirin, Tums, Lipitor, Senokot, Motrin, ibuprofen, Maalox, Ex-Lax, Benadryl, Sudafed, albuterol, Preparation H, MMR vaccine, Claritin, Zoloft, Prilosec OTC, and azithromycin.”
**94**	1	17	2	keto	"lipitor", "got", "im", "staten", "cholesterol", "2017", "january", "mg", "muscle", "wanted"	You look great! My cholesterol is high now too. My endocrinologist wants me to go on Lipitor but after reading info with the prescription, muscle loss is a side effect. I have enough trouble meeting my protein goals as it is and

© 2023 Somani S et al. *JAMA Network Open.*

						take supplemental Mg and Potassium to stave off muscle cramps, so its going to stay on the shelf for now.
**95**	4	12	3	conspiracy	"ivermectin", "covid19", "virus", "showing", "sarscov2", "viral", "x000d", "antiviral", "review", "respiratory"	Review of the antiviral effects of ivermectin. The absolute risk reduction from: The Pfizer injection = 0.7% The Moderna injection = 1.1%. Ivermectin = 9.7% Sources: Meta analysis, Ivermectin lowers death rates by 62.0% RCT 114 patients in Egypt, 57 treated with ivermectin mucoadhesive nanosuspension intranasal spray, showing faster recovery and viral clearance with treatment. Review of ivermectin trials and epidemiological data, concluding that ivermectin is effective for prophylaxis and treatment, and should be globally and systematically deployed in the prevention and treatment of COVID-19. Safety study concluding that ivermectin was generally well tolerated, with no indication of associated CNS toxicity for doses up to 10 times the highest FDA-approved dose. Adverse effects were similar between ivermectin and placebo and did not increase with dose. Authors also show that the plasma concentration is much higher when taken with food (geometric mean AUC 2.6 times higher). Antiviral effects have been reported for Zika, dengue, yellow fever, West Nile, Hendra, Newcastle, Venezuelan equine encephalitis, chikungunya, Semliki Forest, Sindbis, Avian influenza A, Porcine Reproductive and Respiratory Syndrome, Human immunodeficiency virus type 1, and severe acute respiratory syndrome coronavirus 2. Small trial of hospitalized patients with 16 of 87 patients being treated with ivermectin showing a significantly lower mean hospital stay with ivermectin Case study of 100 patients treated with ivermectin and doxycycline, with no ICU admission, deaths, or serious side effects reported. Report on 33 patients with persistent or post-acute symptoms treated with ivermectin, showing a high rate of clinical improvement. Panel review of ivermectin reporting that "ivermectin in the dose of 12mg BD alone or in combination with other therapy for 5–7 days may be considered as safe therapeutic option for mild moderate or severe cases of Covid-19 infection. Retrospective 148 hospitalized patients showing triple therapy with ivermectin atorvastatin N-acetylcysteine resulted in a 1.35% case fatality rate which was well below the national average. Ivermectin shortens durantions of symptoms from 10 days to 3 days. Review suggesting that ivermectin may be

© 2023 Somani S et al. *JAMA Network Open.*

						useful for late stage COVID-19. Authors note that ivermectin, in doses at or modestly above the standard clinical dose, may have important clinical potential for managing disorders associated with life-threatening respiratory distress and cytokine storm, such as advanced COVID-19. Ivermectin lowered deaths by 87.9% in a study from Argentina Retrospective study of 115 ivermectin patients and 133 control patients showing significantly lower death and faster viral clearance with Ivermectin A study showing that ivermectin is capable of interfering in different key steps of the SARS-CoV-2 replication cycle. RCT for ivermectin doxycycline showing improvements in mortality, recovery, progression, and virological cure. How does it work? The mechanisms of action of Ivermectin against SARS-CoV-2: An evidence-based clinical review article The FDA-approved drug ivermectin inhibits the replication of SARS-CoV-2 in vitro Ivermectin is an inhibitor of the COVID-19 causative virus (SARS-CoV-2) in vitro. A single treatment able to effect ~5000-fold reduction in virus at 48 h in cell culture. Ivermectin as a Broad-Spectrum Host-Directed Antiviral: The Real Deal? Excitingly, cell culture experiments show robust antiviral action towards HIV-1, dengue virus (DENV), Zika virus, West Nile virus, Venezuelan equine encephalitis virus, Chikungunya virus, Pseudorabies virus, adenovirus, and SARS-CoV-2 (COVID-19). Ivermectin for COVID-19: real-time meta analysis of 60 studies • Meta analysis using the most serious outcome reported shows 76% and 85% improvement for early treatment and prophylaxis (RR 0.24 [0.14-0.41] and 0.15 [0.09-0.25]), with similar results after exclusion based sensitivity analysis, restriction to peer-reviewed studies, and restriction to Randomized Controlled Trials. • 81% and 96% lower mortality is observed for early treatment and prophylaxis (RR 0.19 [0.07-0.54] and 0.04 [0.00-0.58]). Statistically significant improvements are seen for mortality, ventilation, hospitalization, cases, and viral clearance. 28 studies show statistically significant improvements in isolation. Ivermectin has been found to reduce COVID-19 mortality by 81% It's also safe, inexpensive and widely available, with decades of clinical usage suggesting it has a "high margin of safety." In one trial, 58 volunteers took 12 milligrams of ivermectin once per month for four

© 2023 Somani S et al. *JAMA Network Open.*

						months. Only four (6.96%) came down with mild COVID-19 symptoms during the May through August 2020 trial period. In comparison, 44 of 60 health care workers (73.3%) who had declined the medication were diagnosed with COVID-19. If you were to say, tell me the characteristics of a perfect drug to treat COVID-19, what would you ask for?' he [Marik] said. 'I think you would ask firstly for something that's safe, that's cheap, that's readily available, and has anti-viral and anti-inflammatory properties. People would say, 'That's ridiculous. There could not possibly be a drug that has all of those characteristics. That's just unreasonable. But we do have such a drug. The drug is called Ivermectin.' If it was universally distributed at a dose that costs ten American cents in India and about the cost of a Big Mac in the United States, he said, Ivermectin would save countless lives, crush variants, eliminate the need for endless big pharma booster shots, and end the pandemic all over the world."
**96**		16	2	keto	"yeast", "rice", "red", "supplement", "coq10", "costco", "tablets", "atherosclerosis", "try", "natural"	If your doctor recommended a statin, I think you should give it a try. He is right about how some people just produce more cholesterol, and they are proven to reduce the risks of stroke and heart attacks. If you happen to be diabetic, it's even more important. You can always try a low dose statin if you are reluctant, or you can talk to your doctor about a Red Yeast Rice supplement. Best of luck to you!
**97**		9	6	Supplements	"ryr", "lovastatin", "monacolins", "fda", "monacolin", "yeast", "product", "supplements", "cafeine", "rice"	Unfortunately, in the US at least: It appears that the FDA (American) has banned sales of any RYR product with 'non-negligible' levels of Monacolin K/Lovastatin due to the position of Lovastatin as a pharmaceutical drug; this significantly reduces the supposed benefits associated with red yeast extract *[source](It's possible that certain producers of RYR are 'sneaking' (intentionally or accidentally) product to market that has higher levels of Monacolin K/Lovastatin. But I would assume that most RYR supplements (especially from the bigger, more trustworthy producers) are neutered and thus less useful as an OTC natural statin.
**98**		4	3	science	"favorably", "curcumin", "turmeric", "abstracts", "additionalcurcumin", "published", "oxaliplatin",	Given the sheer density of research performed on this remarkable spice, it is no wonder that a growing number of studies have concluded that it compares favorably to a variety of conventional medications, including: *

© 2023 Somani S et al. *JAMA Network Open.*

					"spice", "imipramine", "lipitoratorvastatincholesterol"	Lipitor/Atorvastatin(cholesterol medication) * Corticosteroids (steroid medications) * Prozac/Fluoxetine & Imipramine (antidepressants) * Aspirin (blood thinner) * Anti-inflammatory Drugs * Oxaliplatin (chemotherapy drug) * Metformin (diabetes drug)
**99**		4	6	ScientificNutrition	"supplement", "nissen", "supplements", "district", "dietary", "natural", "red", "appropriately", "willow", "yeast"	I thought this was a little bit ironic Nissen places a substantial portion of the blame for statin denialism on the Dietary Supplement Health and Education Act of 1994 (DSHEA), which loosened regulation of dietary supplements, spurring what is now a $30 billion a year industry selling an “array of worthless or harmful dietary supplements.” According to Nissen, supplement makers “commonly imply benefits that have never been confirmed in formal clinical studies.” As I remember it, statins were in fact derived from a natural health supplement called red rice yeast extract, and somehow the drug companies were able to patent the natural molecule and derivatives thereof in spite of prior use in this application. Not that I would trust the supplement over the prescription, due to standardisation and less contaminants. But you could technically have kept it as a natural product. People like Nissen bitch about supplements but at the same seem to attack the industry when it wants to standardise its pills they complain it is unfair to pharmaceutical companies and these supplements should now be regulated as drugs.
**100**		4	6	todayilearned	"compounds", "fungi", "zebrafish", "fungal", "biological", "embryos", "thousand", "active", "ai", "biologically"	Four humors is Ancient Greek medicine. By far some traditions don’t stand the test of time. Ayruveda (TIM), Traditional Chinese Medicine is still practiced today. And “alternative” medicine is the name that’s given from a western standpoint or bias. For many cultures traditional medicine is medicine. They have antibacterial agents, anticoagulants, analgesics. It still is used today in remote areas. There’s a whole study of plant medicine called pharmacognosy, which as I mention earlier is a part of drug discovery. Look at over the counter active ingredients. Eugonol tooth ache? (Clove bud) Methyl Salicylate for back ache or sports? (Wintergreen) 1,8 cineole aka eucalyptus in Vicks vapor rub? (Eucalyptus) That’s not even bringing up plants like opium, digitalis, cannabis. When they discovered statin drugs for cholesterol, you look back at ancient Chinese remedies for cardiovascular health and find fermented red yeast rice over 1000 years ago

© 2023 Somani S et al. *JAMA Network Open.*

						which can actually create statin compounds. What are the odds of that a happening by sheer coincidence and continue to use it for thousands of years. A broken clock is right twice a day, is 1 out of 720 chance. The chance that a person chooses red yeast rice for blood not knowing about statin over thousand years ago or a person with anti malaria medicine over a thousand years ago and the plant happen to have the compounds that people only recently discover is uncanny. That's why it's studied. Some ingredients make sense, some don't but the fact their remedies help must be part of trial and error and some believe divine or intuition.

© 2023 Somani S et al. *JAMA Network Open.*

eTable 2. Mean Sentiment Across Subreddits

Mean of the average sentiment score for all discussions by subreddit. Values closer to -1 reflect negative sentiment, values closer to 0 reflect neutral sentiment, and values close to 1 reflect positive sentiment.

Subreddit	Mean Sentiment
keto	-0.31
Cholesterol	-0.17
diabetes	-0.21
science	-0.31
ketoscience	-0.36
nutrition	-0.31
ScientificNutrition	-0.30
news	-0.45
todayilearned	-0.37
conspiracy	-0.52
Supplements	-0.25
Health	-0.36
PlantBasedDiet	-0.15
askscience	-0.22
COVID19	-0.12
Paleo	-0.38
longevity	-0.17
skeptic	-0.40
stopusingstatins	0.00

© 2023 Somani S et al. *JAMA Network Open.*
