# Supplementary material for: Artificial Intelligence–Enabled Analysis of Statin-Related Topics and Sentiments on Social Media
Source: JAMA Netw Open. 2023 Apr 24;6(4):e239747. doi: 10.1001/jamanetworkopen.2023.9747 (PMC10126874; doi:10.1001/jamanetworkopen.2023.9747)
Supplement: Supplement 2. — Data Sharing Statement [file jamanetwopen-e239747-s002.pdf]

Data Sharing Statement

Somani. Artificial Intelligence-Enabled Analysis of Statin-Related Topics and Sentiments on Social Media. *JAMA Netw Open*. Published April 24, 2023.
doi:10.1001/jamanetworkopen.2023.9747

Data
Data available: Yes
Data types: Data (not involving human participants)
How to access data: Data will be available on Stanford github and upon request to the investigators
When available: With publication

Supporting Documents
Document types: Statistical/analytic code
How to access documents: Stanford github
When available: With publication

Additional Information
Who can access the data: Anyone requesting the data since the data is openly available
Types of analyses: n/a
Mechanisms of data availability: Link to the code will be provided. Data is publicly available and can be downloaded by other research teams
